# Ganglioside GD2 Contributes to a Stem‐Like Phenotype in Intrahepatic Cholangiocarcinoma

**DOI:** 10.1111/liv.16208

**Published:** 2024-12-26

**Authors:** Antonella Mannini, Mirella Pastore, Alessia Giachi, Margherita Correnti, Elena Spínola Lasso, Tiziano Lottini, Benedetta Piombanti, Ignazia Tusa, Elisabetta Rovida, Cédric Coulouarn, Jesper B. Andersen, Monika Lewinska, Claudia Campani, V. Lokesh Battula, Bin Yuan, Massimo Aureli, Emma V. Carsana, Caterina Peraldo Neia, Paola Ostano, Alessia Tani, Daniele Nosi, Anna Vanni, Laura Maggi, Luca Di Tommaso, Giuseppina Comito, Stefania Madiai, Annarosa Arcangeli, Fabio Marra, Chiara Raggi

**Affiliations:** ^1^ Department of Experimental and Clinical Medicine University of Florence Florence Italy; ^2^ Department of Biomedical Sciences for Health University of Milan Milan Italy; ^3^ Department of Experimental and Clinical Biomedical Sciences ‘Mario Serio’ University of Florence Florence Italy; ^4^ Univ Rennes, Inserm, Inra Institut NUMECAN (Nutrition Metabolisms and Cancer)‐UMR_S 1241, UMR_A 1341 Rennes France; ^5^ Biotech Research and Innovation Centre University of Copenhagen Copenhagen Denmark; ^6^ Division of Cancer Medicine, Department of Leukemia The University of Texas MD Anderson Cancer Center Houston Texas USA; ^7^ Department of Medical Biotechnology and Translational Medicine University of Milan Milan Italy; ^8^ Fondazione Edo Ed Elvo Tempia Valenta Biella Italy; ^9^ Humanitas Research Hospital‐IRCCS, Pathology Unit Rozzano Italy

**Keywords:** cancer stem cells, cancer stemness biomarker, GD2 ganglioside, GD3 synthase (o ST8SIA1), intrahepatic cholangiocarcinoma

## Abstract

**Background & Aims:**

GD2, a member of the ganglioside (GS) family (sialic acid‐containing glycosphingolipids), is a potential biomarker of cancer stem cells (CSC) in several tumours. However, the possible role of GD2 and its biosynthetic enzyme, GD3 synthase (GD3S), in intrahepatic cholangiocarcinoma (iCCA) has not been explored.

**Methods:**

The stem‐like subset of two iCCA cell lines was enriched by sphere culture (SPH) and compared to monolayer parental cells (MON). GS profiles were evaluated by chromatography, after feeding with radioactive sphingosine. Membrane GD2 expression was evaluated by FACS, and the expression of enzymes of GS biosynthesis was analysed by RT‐qPCR. The modulation of stem features by GS was investigated in vitro and in vivo using GD3S‐overexpressing cells and corroborated by global transcriptomic analysis.

**Results:**

GS composition was markedly different comparing SPH and MON. Among complex GS, iCCA‐SPH showed increased GD2 levels, in agreement with the high expression levels of GD3 and GM2/GD2 synthases. iCCA cells overexpressing GD3S had higher sphere‐forming ability, invasive properties and drug resistance than parental cells. NOD/SCID mice implanted with CCLP1 cells overexpressing GD3S developed larger tumours than control cells. By global transcriptomic analysis, ontology investigation identified 74 processes shared by the iCCA‐SPH and GD3S‐transfected cells, with enrichment for development and morphogenesis processes, MAPK signalling and locomotion. In a cohort of patients with iCCA, GD3S expression was correlated with lymph node invasion, indicating a possible relevance of GD3S in the clinical setting.

**Conclusions:**

The profile of GS derivatives regulates the stem‐like properties of iCCA cells.

**Abbreviations**
CSCs: cancer stem cells
EMT: epithelial to mesenchymal transition
GD3S: GD3 synthase
GM2/GD2S: GM2/GD2 synthase
GS: gangliosides
GSEA: gene set‐enrichment analysis
iCCA: intrahepatic cholangiocarcinoma
MON: monolayer
PPMP: D‐threo‐1‐Phenyl‐2‐palmitoylamino‐3‐morpholino‐1‐propanol
SPH: sphere cultures

**Summary**

- Gangliosides, specifically GD2, contribute to cholangiocarcinoma progression. Elevated GD2 expression, observed in 3D cultures, correlates with increased aggressiveness and drug resistance.
- Furthermore, GD2 upregulation promotes tumour growth in vivo. Overexpression of GD3 synthase (GD3S), the enzyme responsible for GD2 synthesis, mirrors these effects.
- These findings implicate both GD2 and GD3S as potential therapeutic targets in cholangiocarcinoma.

## 1 Introduction

Cholangiocarcinoma (CCA) is a heterogeneous and aggressive cancer arising from malignant transformation within the biliary tree. This tumour represents the second most frequent type of primary liver cancer after hepatocellular carcinoma. The severity of CCA and the limited benefit of current therapeutic strategies make this disease a top priority in the field of cancer research, especially for tumours arising in the intrahepatic bile ducts (iCCA), which is less common but rapidly rising in incidence in Western countries [1, 2]. Cancer stem cells (CSC) are considered the driving force for tumour initiation, development, spread, recurrence and resistance to chemoradiotherapy in many solid tumours, including intrahepatic CCA (iCCA) [3, 4, 5]. In CCA, the presence of cells with characteristics of CSC has been correlated with more aggressive tumour characteristics and poorer patient prognosis [3]. Identification of the roles of CSC in iCCA and in other solid tumours has been favoured by the development of in vitro systems enriched in stem‐like cells. In particular, we and others have developed the functional assay of 3D tumour sphere (SPH) formation, as an efficient in vitro system to enrich and maintain cells with stem‐like properties [3].

Over the years, the ganglioside class (GS) has received attention for its unexpected role in cancer stemness [6, 7, 8, 9]. GS are glycosphingolipids consisting of a ceramide backbone and a combination of glucose, galactose and N‐acetylgalactosamine with N‐acetylneuraminic acid (sialic acid). GM3 (ceramide–glucose–galactose–sialic acid) is the precursor of all other GS, obtained through the sequential addition of other carbohydrate groups and sialic acid catalysed by specific glycosyl‐ and/or sialyl‐transferases [10, 11, 12] (Scheme S1). In cancer promotion and cancer stemness, the ganglioside GD2 has received specific attention, together with GD3 synthase (GD3S), the enzyme which synthesises GD3, the GD2 precursor [13, 14, 15, 16]. However, although GS composition has been studied in different tumour types, no evidence of the implication of these molecules in iCCA is available, especially in the stem cell compartment. Starting from the study of GS patterns in iCCA lines, here we show that GD2 and GD3S are involved in the biology of the iCCA stem‐like compartment, possibly supporting tumour fate and CSC formation.

## 2 Results

### 2.1 GS Profile Is Altered in the Stem‐Like Subset of iCCA

The development of in vitro systems that enrich for stem‐like cells, such as three‐dimensional (3D) tumour sphere (SPH) formation, has significantly aided research on CSC. As previously demonstrated by our group, SPH exhibit an overrepresentation of stemness‐related features compared to monolayer cultures (MON) of the parental cells [3, 4, 5] (Figure S1).

**FIGURE 1:**
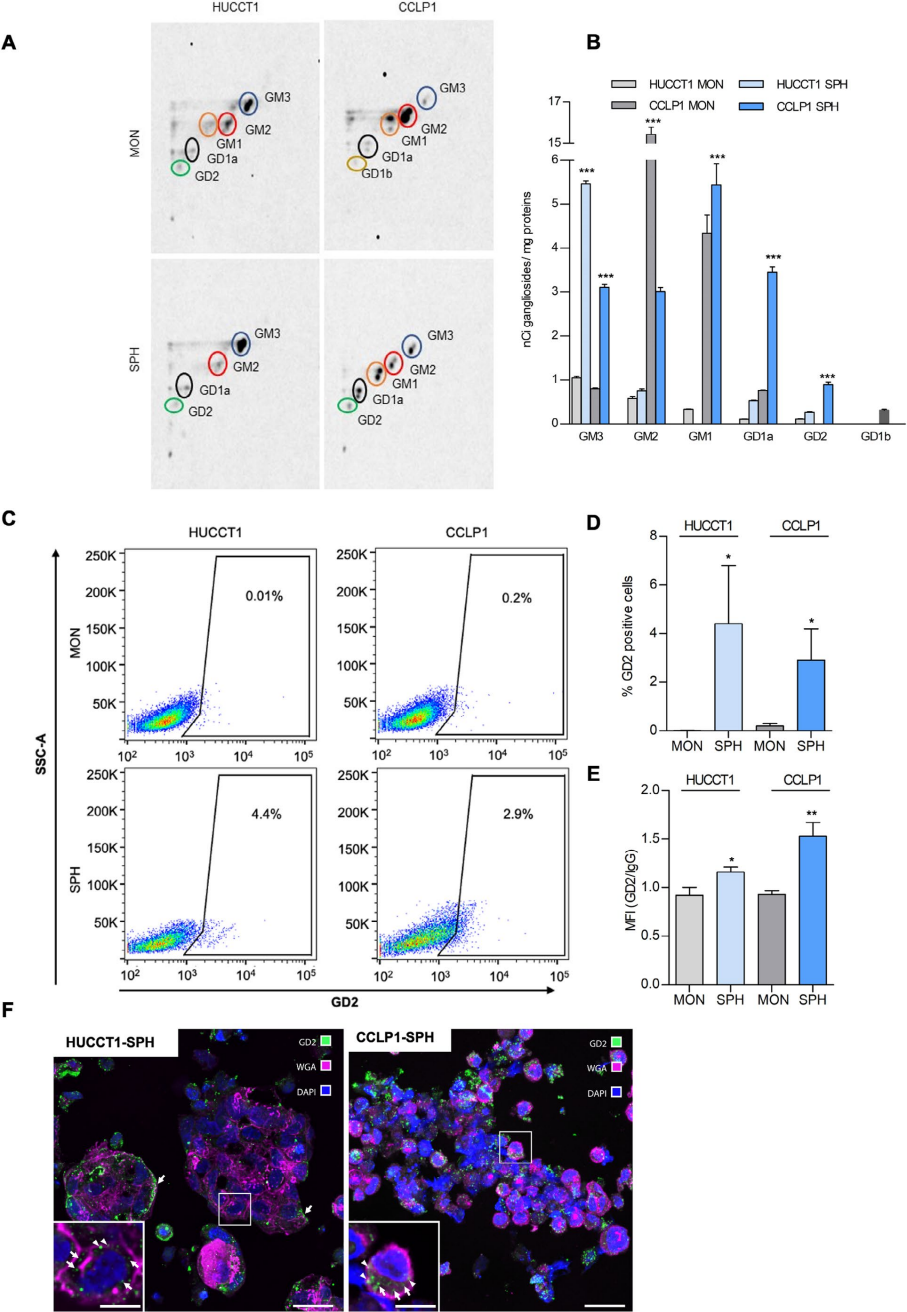
Ganglioside patterns of iCCA‐MON and ‐SPH. (A) Representative digital autoradiography of ganglioside pattern and (B) quantification of the radioactivity associated with individual gangliosides in HUCCT1 and CCLP1 (MON and SPH), fed with radioactive sphingosine in order to label at the steady state cell sphingolipids. Data are expressed as nCi of ganglioside/mg of cell proteins, as mean ± SEM (*n* = 3, ***p < 0.001, *t*‐test SPH vs. MON). (C) FACS analysis strategy. Representative dot plots foretection GD2 ganglioside in iCCA‐MON and ‐SPH.Graph bar reported as (D) percent (%) of positive cells and (E) MFI values (*p < 0.05, **p < 0.01 *t*‐test, SPH vs. MON). (F) Representative confocal immunofluorescence images of sections of OCT‐embedded SPH of human HUCCT1 and CCLP1 cells immunostained to reveal GD2 (green). Cell plasma membranes and nuclei were counterstained with WGA Alexa 555 conjugated (magenta) and DAPI (blue), respectively; scale bars = 20 μm. Insets show high‐resolution details of the areas highlighted in the respective figures; individual optical sections acquired inside the cells display the localisation of GD2 on the cell membrane (arrowheads) and in the cytoplasm (arrows); scale bars = 7 μm.

We first compared the GS composition in iCCA lines, to evaluate possible changes associated with the stem‐like subset. In HUCCT1‐MON, monosialo‐GS, such as GM3 or GM2, were represented, whereas CCLP1‐MON contained less GM3, and very high levels of GM2 and GM1 gangliosides (Figure 1A,B). The GS subclasses in the 3D SPH cultures showed a profile more pronounced in the monosialo‐GS (Figure 1A,B). Analysis of the stem‐like compartment indicated that in HUCCT1‐SPH GM3 was increased and associated with a small, non‐significant increase in more complex GS (Figure 1A,B). In contrast, in CCLP1‐SPH both monosialo‐ and disialo‐GS were markedly increased, and ganglioside GD2 accounted for 4.4% of GD2^pos^ CCLP1‐SPH, while it was absent in their MON parental (Figure 1A,B).

We also analysed the presence of GD2 on the external layer of the plasma membrane. Compared to MON, in both iCCA lines small but discrete percentages of GD2^**pos**^ cells were detected in SPH, associated with a significant increase in mean fluorescent intensity (MFI), especially in CCLP1 (Figure 1C–E). Because external membrane GD2 presence in SPH was relatively low when assessed by FACS, we performed additional experiments using immunofluorescence on sections of CCLP1 and HUCCT1 spheres. While no apparent difference in GD2 expression was observed between the outer and inner SPH‐layers, notable intracellular GD2 accumulation was detected. Confocal microscopy revealed heterogeneous presence of GD2 in SPH of both human HUCCT1 and CCLP1 cells suggesting that GD2 is not limited to the external membrane; it is also present intracellularly, associated with other intracellular membranes (Figure 1F). In the HUCCT1 samples, SPH consisted of cells tightly adhered to each other and the GD2 signal was often concentrated in the outermost cell layers, in the portions of the membrane not involved in cell contacts (arrows). Interestingly, in both samples, intracellular expression of GD2 was also evident, possibly on the inner membranes (insets in Figure 1F) suggesting a potential role of intracellular trafficking in GD2 formation and expression.

**FIGURE 2:**
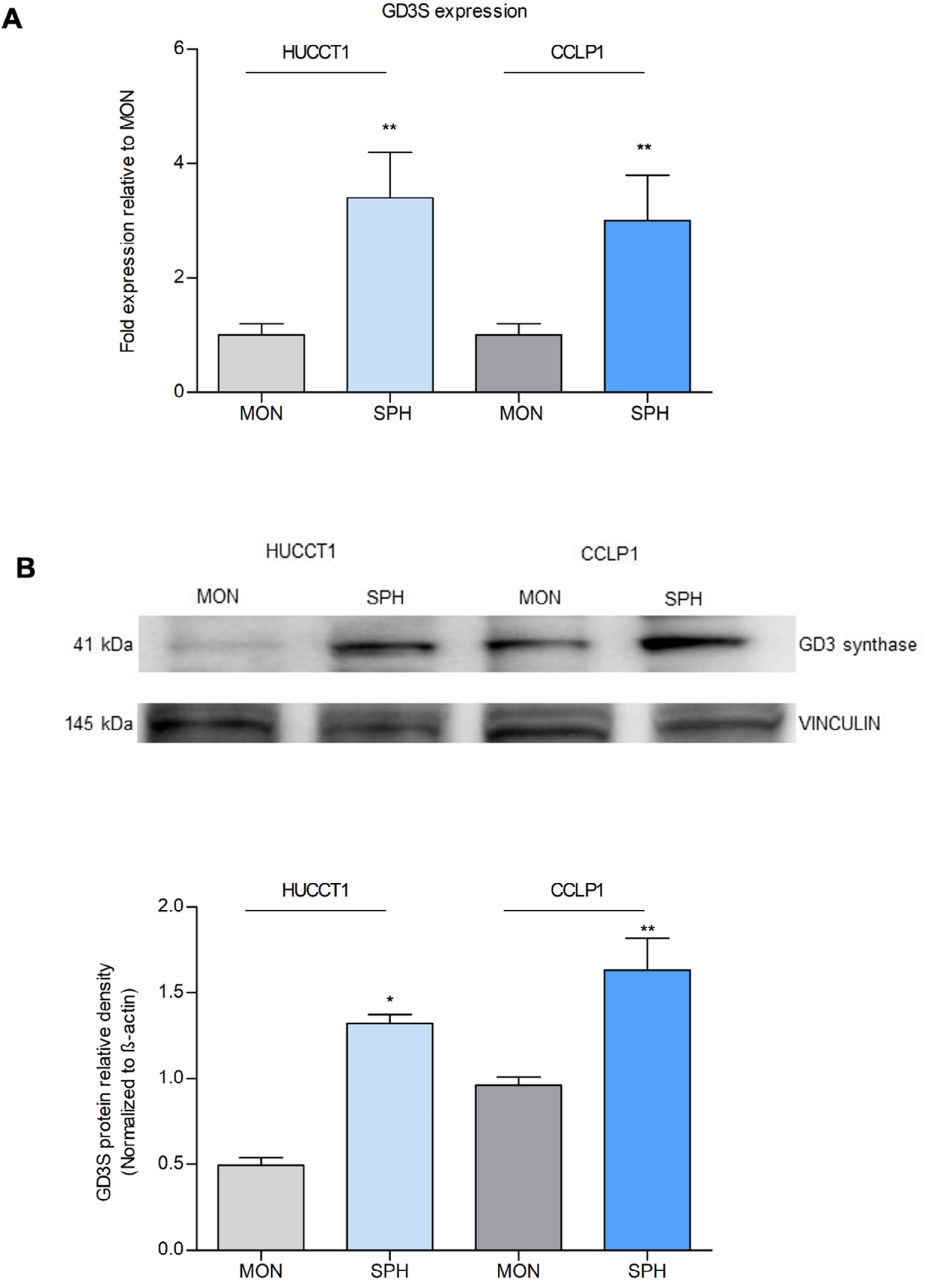
Expression of GD3 synthase in iCCA‐MON and ‐SPH. (A) The GD3 synthase was evaluated in iCCA cells (HUCCT1 and CCLP1) and reported as fold expression relative to MON (*n* = 5, **p < 0.01 SPH vs. MON). (B) Western blot of the protein levels of GD3S were established in MON and SPH of the iCCA cells. Vinculin immunoblot was performed to ensure equal loading. Densitometry of GD3S/Vinculin expression (*n* = 3) was shown in the graph (*p < 0.05, **p < 0.001, *t*‐test SPH vs. MON).

The specific transferases involved in GD2 synthesis were also evaluated. Expression of the GD3S gene was more than twofold increased in SPH of both iCCA lines, compared to the MON (Figure 2A). These findings were confirmed at the protein level (Figure 2B). In addition, the expression of GM2/GD2S, involved in the biosynthesis of GM2 and GD2, was strongly expressed in both iCCA‐SPH (Figure S2).

**FIGURE 3:**
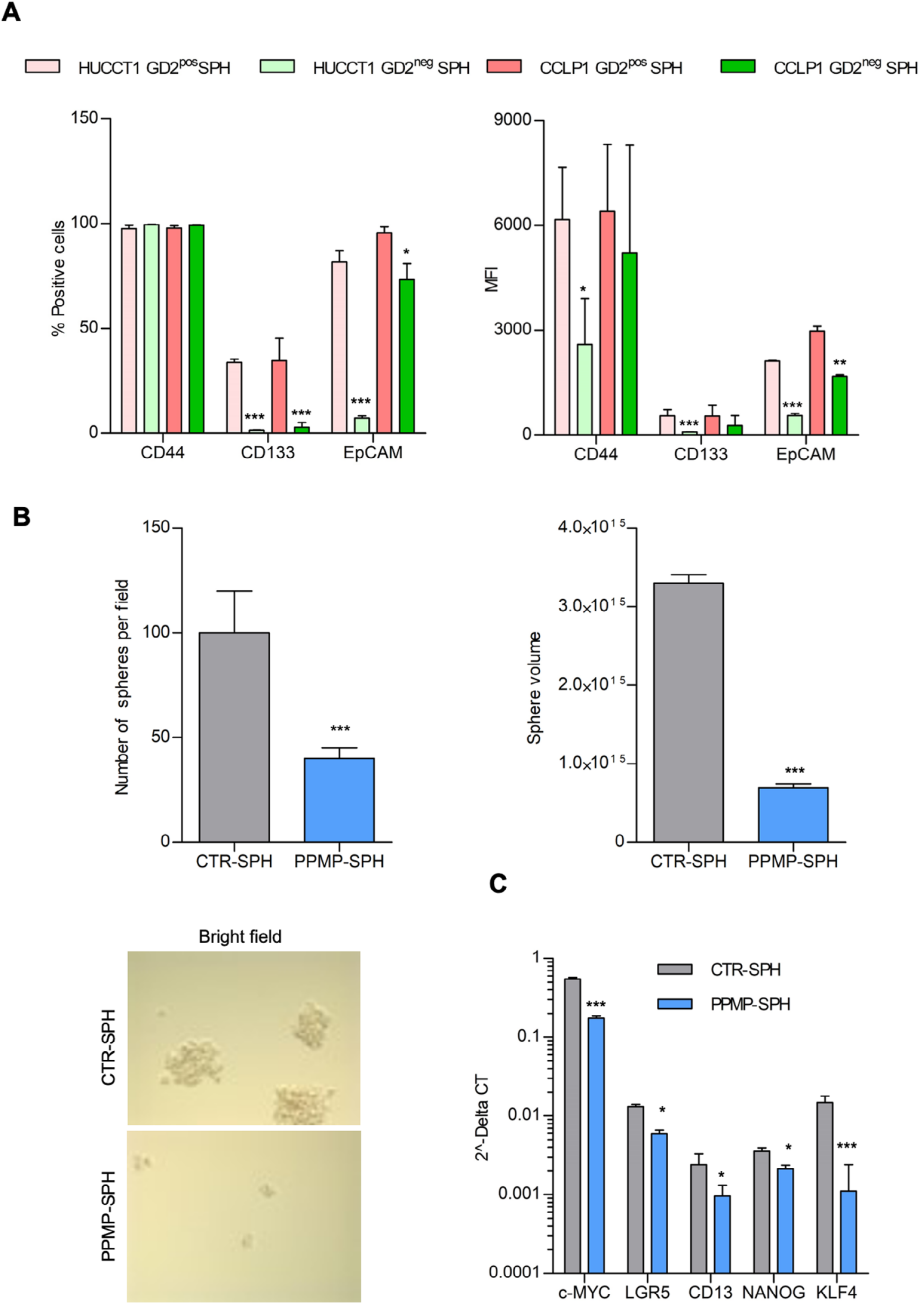
Evidence for association between GD2 and stem‐like features. (A) Evaluation of stem‐like surface proteins in GD2^pos^ and GD2^neg^ SPH cells by FACS. reported as percentage of positive cells and MFI. (B) iCCA sphere‐forming efficiency and sphere‐volume in CCLP1 cells after PPMP exposure. Mean ± SEM (*n* = 3, ***p ≤ 0.001 PPMP‐SPH vs. CTR‐SPH). (C) Impact of PPMP on the expression of several stem‐like genes. The mRNA expression of stem‐like genes is presented as 2^deltaCT. Data are mean ± SEM (*n* = 3, *p ≤ 0.05, ***p ≤ 0.001 PPMP‐SPH vs. CTR‐SPH).

To provide additional evidence for the association between discrete ganglioside classes and cell stemness, CCLP1‐SPH were sorted based on the membrane expression of GD2. GD2^pos^ cells expressed at significantly higher levels several biomarkers related to pluripotency (e.g., KLF‐4, SOX‐2), drug resistance (ABCG2), and stemness (CD133), compared to CCLP1‐SPH GD2^neg^ (Figure S3). Notably, GD2^**pos**^‐SPH of both iCCA lines showed a much higher percentage of positive cells and MFI relative to stem‐like markers such as CD44, CD133 and EpCAM compared to GD2^**neg**^‐SPH (Figure 3A).

### 2.2 Changes in the Expression of GS Synthases Modulate Stem‐Like Features of iCCA Cells

We next explored the biologic significance of increased GD2 expression on the surface of stem‐like iCCA cells, employing two complementary strategies. First, we analysed the effects of PPMP (D‐threo‐1‐Phenyl‐2‐palmitoylamino‐3‐morpholino‐1‐propanol), a specific inhibitor of glucosylceramide synthase. In the presence of PPMP, CCLP1‐SPH showed a significant reduction in sphere‐forming ability and sphere volume (Figure 3B), as well as in the expression of genes related to stemness pluripotency or self‐renewal (c‐MYC, KLF‐4, CD13, NANOG, LGR5, Figure 3C).

**FIGURE 4:**
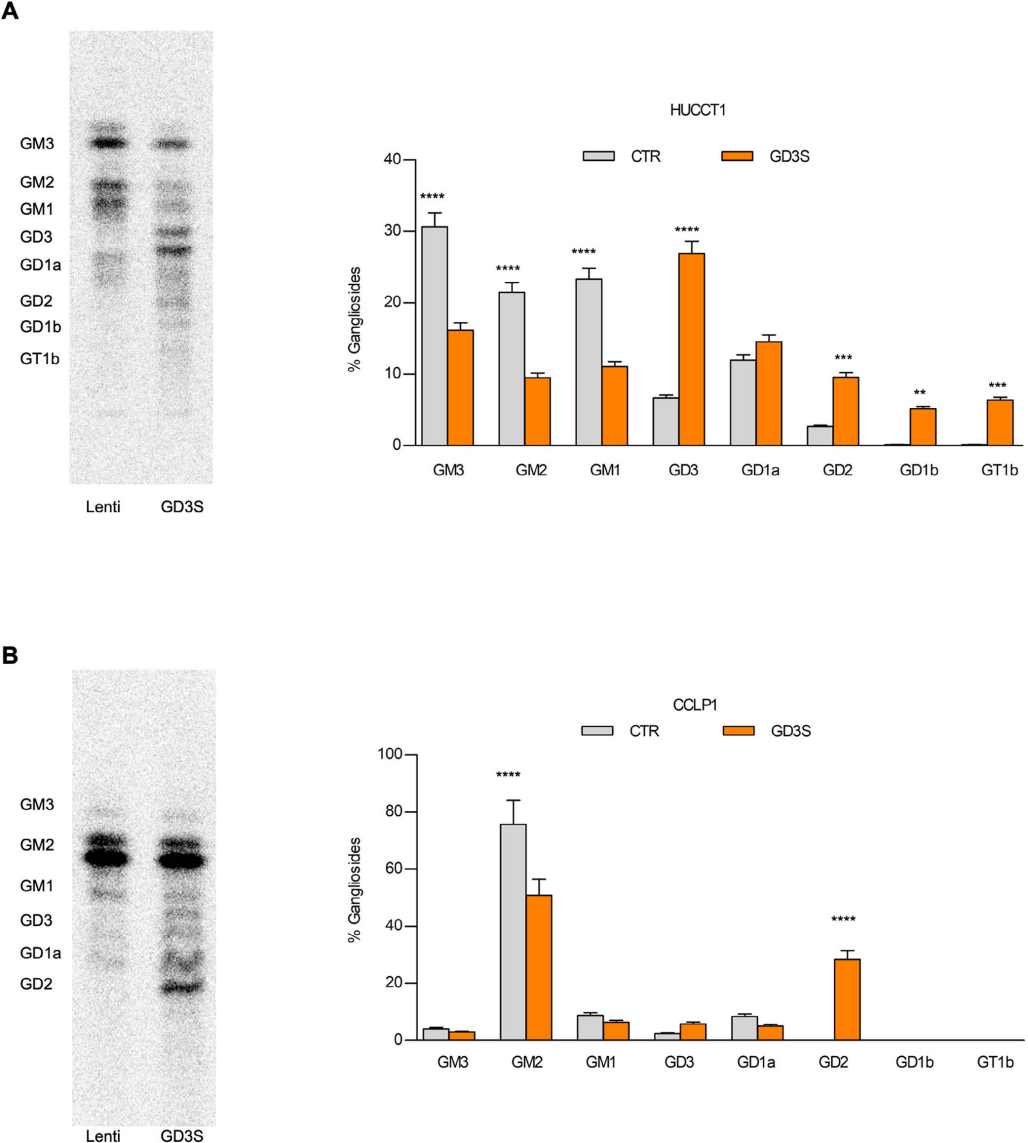
Ganglioside patterns of iCCA cells overexpressing GD3S. (A) Representative digital autoradiography of ganglioside pattern and quantification of the percentage of individual ganglioside in iCCA‐HUCCT1 overexpressing or not (CTR) GD3S after feeding with radioactive sphingosine in order to label at the steady state cell sphingolipids. Data are expressed as % of ganglioside; Mean ± SEM (*n* = 3, ***p < 0.001, one‐way ANOVA Lenti vs. GD3S). (B) Representative digital autoradiography of ganglioside pattern and quantification of the percentage of individual ganglioside in iCCA‐CCLP1 overexpressing or not (CTR) GD3S after feeding with radioactive sphingosine in order to label at the steady state cell sphingolipids. Data are expressed as % of ganglioside; mean ± SEM (*n* = 3, *p < 0.05, **p < 0.01, ***p < 0.001, ****p < 0.0001, one‐way ANOVA Lenti vs. GD3S).

While the effects of PPMP are highly suggestive of the involvement of gangliosides in the biology of iCCA CSC, additional experiments were conducted to define the specific role of GD2, transfecting HUCCT1 and CCLP1 cells with an expression vector encoding for GD3S (Figure S4A). The GS profile shifted toward the synthesis of more complex classes, and both GD3S‐transfected iCCA cells showed a pronounced increase in GD2 (Figure 4A,B). In HUCCT1 cells, the GS profile was more generally affected than in CCLP1, whereas this latter cell type showed a more marked increase in GD2 synthesis, without altering the other GS classes. As expected, increased GD3S expression in both GD3S‐transfected iCCA lines was associated with a markedly higher percentage of GD2^**pos**^ cells (82%–99%) evaluated by FACS (Figure S4B).

**FIGURE 5:**
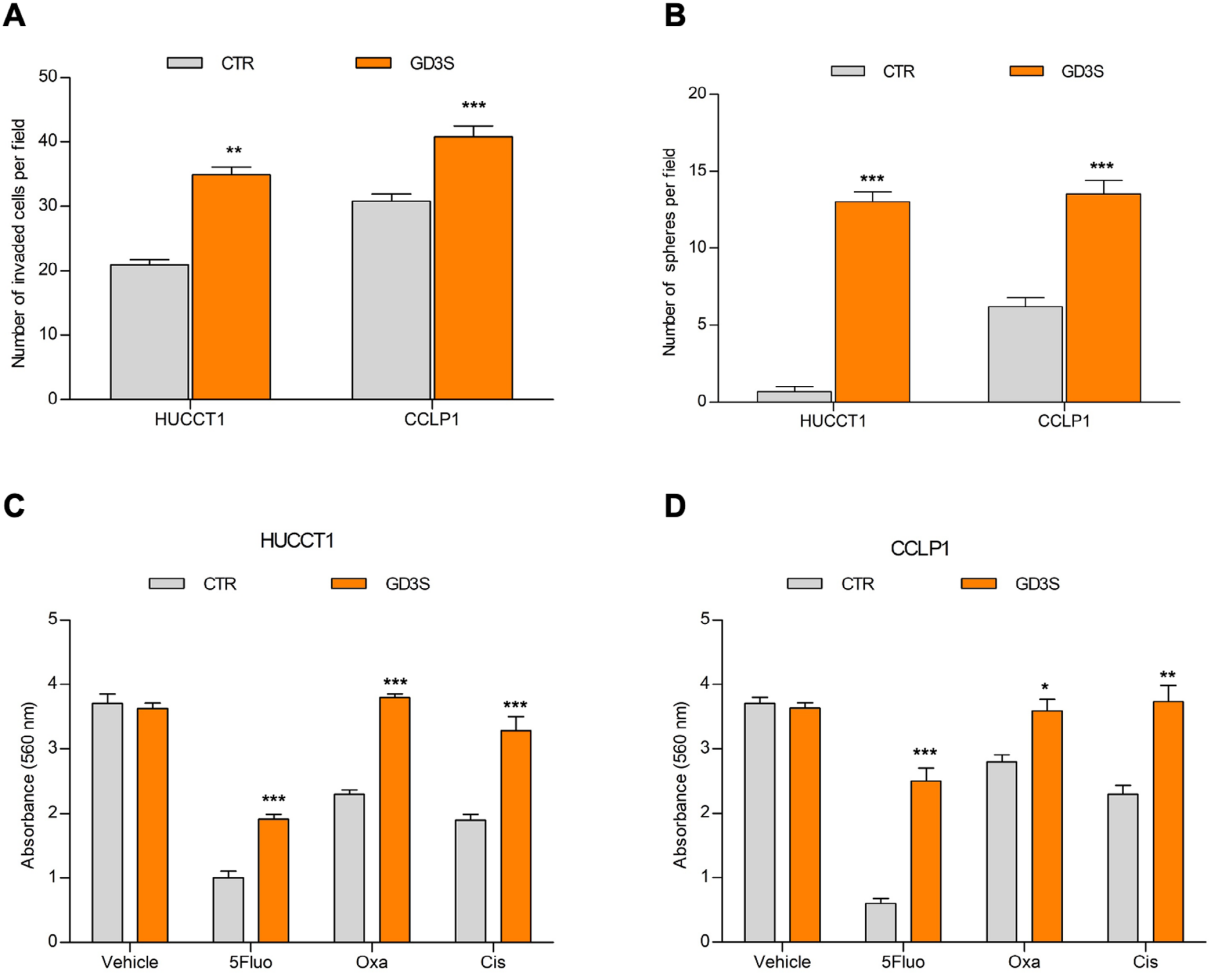
In vitro tumour‐stem‐like properties of iCCA cells overexpressing GD3S. (A) Invasion of CCLP1 and HUCCT1 transfected cells was measured in modified Boyden chambers; mean ± SEM (*n* = 5, **p ≤ 0.01, ***p ≤ 0.001 GD3S vs. CTR). (B) iCCA sphere‐forming efficiency in HUCCT1 and CCLP1 transfected cells. Mean ± SEM (*n* = 3, **p ≤ 0.01, ***p ≤ 0.001 GD3S vs. CTR). (C, D) CCLP1 and HUCCT1 GD3S‐stably transfected cells were treated with 5‐fluorouracil, cisplatin or oxaliplatin (IC_50_ doses and treatment are described in [3]). Cell viability as measured by absorbance intensity (560 nm) was assessed with crystal violet staining. Mean ± SEM (*n* = 3, *p ≤ 0.05, **p ≤ 0.01, ***p ≤ 0.001 GD3S vs. CTR). CTR, transfected control cells; GD3S, cells stably transfected with GD3 synthase.

We next evaluated whether GD3S could have a role in the modulation of the iCCA stem‐like features. In vitro, both HUCCT1 and CCLP1 cells overexpressing GD3S markedly enhanced the capacity to invade a basement‐like membrane (Figure 5A), and their sphere‐forming ability, an indication of self‐renewal potential (Figure 5B). Due to their drug‐resistance properties, cancer stem cells can escape cytotoxicity and survive chemotherapy and radiotherapy. iCCA cells overexpressing GD3S, exposed to drugs used for first‐line treatment of iCCA, were significantly more viable under pharmacological treatment (Figure 5C,D).

**FIGURE 6:**
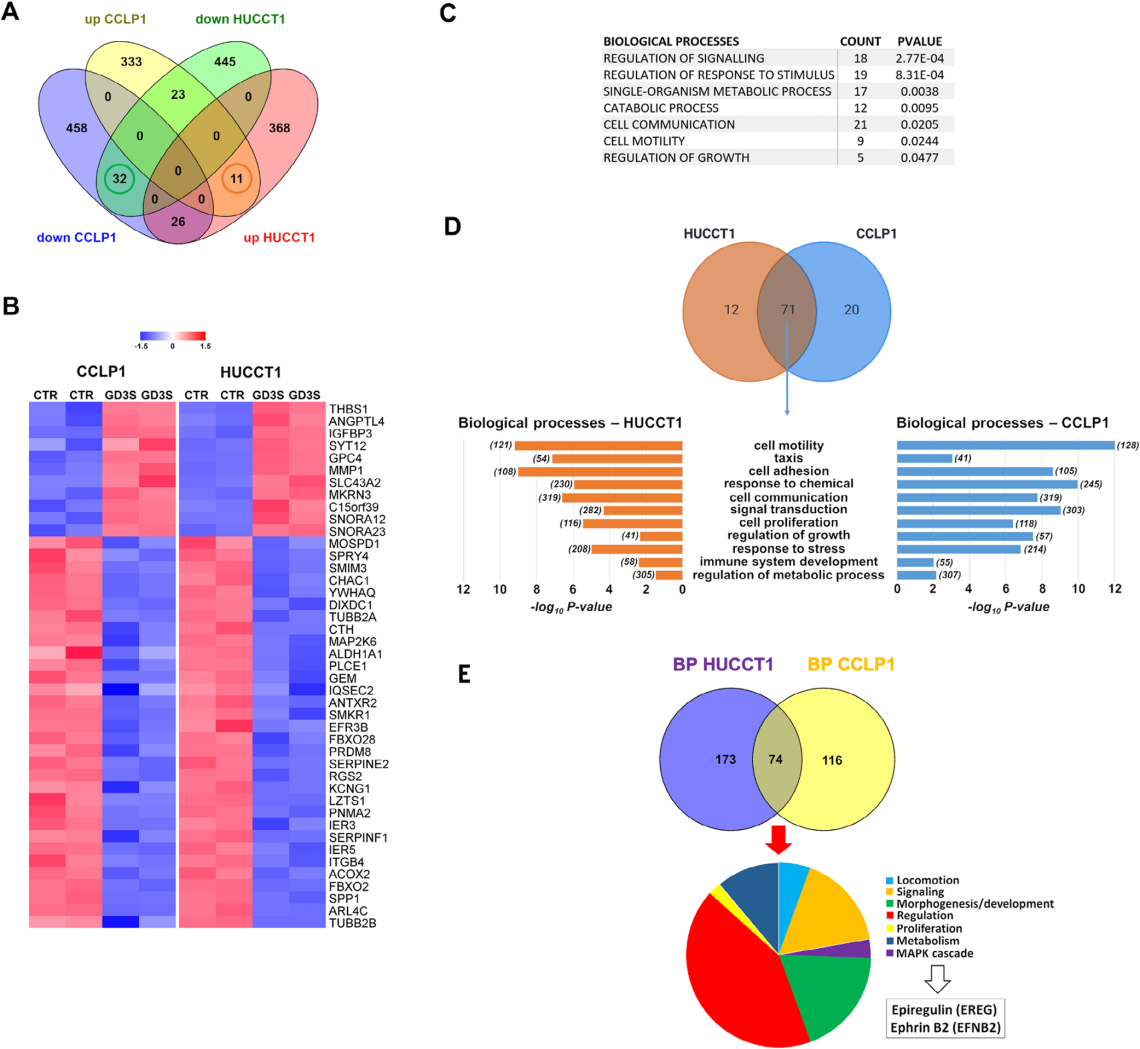
Molecular profile of iCCA cells overexpressing GD3S. (A) Venn diagram of commonly and specific deregulated genes between HUCCT1 and CCLP1 cells transfected with GD3S versus controls. (B) Heatmap of 43 commonly differentially expressed genes in both HUCCT1 and CCLP1 cell lines. Modified *Z* scores of the individual genes, as median‐centred log2 intensity values divided by standard deviation, are shown by a blue‐to‐red gradient variation. (C) Functional enrichment analysis of the 43 commonly deregulated genes. (D) Upper panel: Venn diagram of biological processes (level 2 of the GO) for which the lists of differentially expressed genes in HUCCT1 (orange) and CCLP1 (blue) cells transfected with GD3S versus controls were enriched with a *p* < 0.05. Seventy‐one processes (69%) were commonly enriched in both cell lines. The union of up‐ and down‐regulated genes for the two cell line was used for functional‐enrichment analysis. Lower panel: selection of the non‐redundant processes among the 71 commonly enriched in HUCCT1 (orange) and CCLP1 (blue) cell lines upon GDS3 transfection. Bars represent the negative log base 10 of the *p*. (E) Venn diagram of biological processes obtained by GD3S versus lentiviral and SPH and versus MON comparisons in HUCCT1 (blue) and CCLP1 (yellow). The 74 shared processes were further explored and are represented in the pie chart, with epiregulin and ephrin‐B2 being involved in all the depicted functional classes.

In order to obtain a global view of the genes modulated upon GD3S transfection, we performed a transcriptomic analysis in both iCCA cell lines, selecting the top 1000 up‐ and down‐regulated probes in each CCA cell line (corresponding to 883 and 905 genes in CCLP1 and HUCCT1, respectively) (Figure 6A). From this analysis, 32 genes were down‐regulated and 11 genes up‐regulated in both HUCCT1 and CCLP1 cells (Figure 6A,B). Functional classification of the total list of the 43 deregulated genes revealed an enrichment of biological processes related to signalling, response to stimulus, metabolism, cell communication, motility and growth (Figure 6C). On the other hand, functional enrichment analysis of differentially expressed genes in transfected HUCCT1 and CCLP1 cells versus controls revealed a substantial intersection of statistically significant biological processes from level 2 of the Gene Ontology (*p* < 0.05; Figure 6D). Among the non‐redundant and significant terms in common between HUCCT1 and CCLP1 cells, processes related to cell motility, cell adhesion, cell communication, proliferation, response to stress, immune system development and regulation of metabolic processes were found. The contribution to the enrichment was given by both up‐ and down‐regulated genes upon GD3S transfection in the two cell lines (Figure 6D). In order to disclose possible similarities between GD3S‐transfected cells and spheres, gene ontology analysis was performed. CCLP1 and HUCCT1 transfected with GD3S were compared with parental cells, and a similar analysis was performed comparing SPH with MON. Within the same cell line, among differentially expressed genes, the top 1000 deregulated ones for each comparison (GD3S vs. lentiviral and SPH vs. MON) were selected. We found 151 common deregulated genes in HUCCT1 and 100 in CCLP1. Gene Ontology was performed using these gene lists focusing on biological processes level 5 with a *p* < 0.05. We then identified 74 processes shared by the two cell lines (Figure 6E); as shown, there is an enrichment for development and morphogenesis processes, signalling, in particular, MAPK pathway and locomotion. This is particularly relevant, as genes associated with stem cellness often overlap with those regulating development, differentiation and tissue morphogenesis. Stem cells, by definition, possess the capacity to differentiate into multiple cell types, a process inherently linked to morphogenetic pathways. For instance, genes that control cell fate, tissue organisation and embryonic development are frequently implicated in maintaining stem cell characteristics such as self‐renewal and pluripotency. Thus, it is plausible that the genes we identified in these categories include those that play dual roles in promoting stem cell‐like traits and driving developmental programs (Figures S5–S7).

Finally, all transcriptomic investigations highlighted the constant involvement of epiregulin and ephrin B2, which are involved in cancer progression and tumour cell invasion, respectively. Interestingly, epiregulin and ephrin B2 are expressed in the lipid raft and/or caveolae structures, whose GS are specific constituents.

**FIGURE 7:**
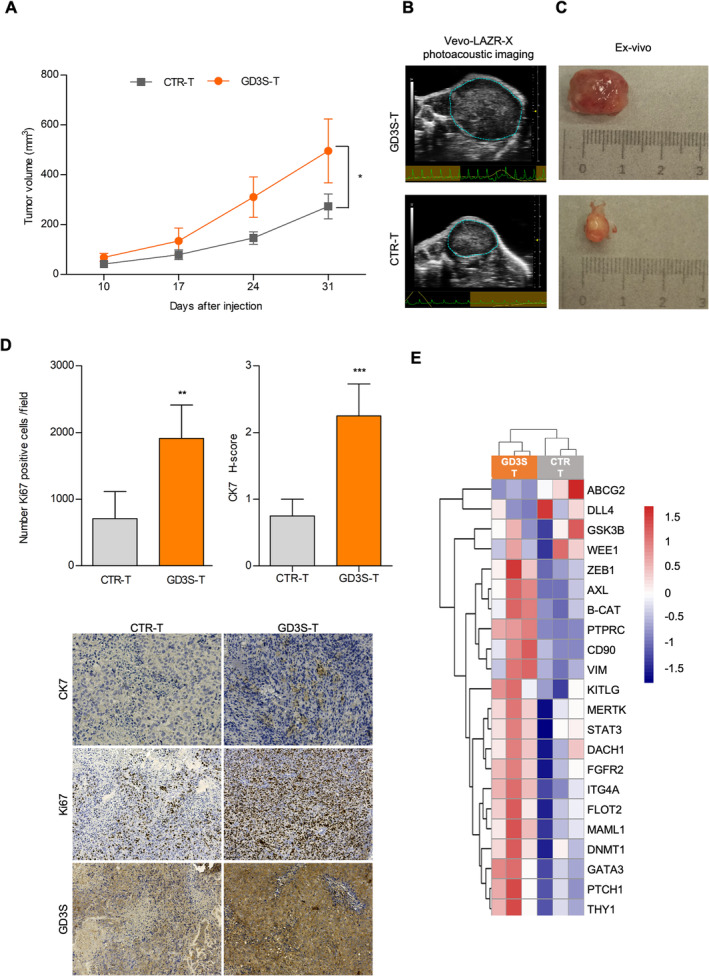
Impact of GD3S in GD3S‐transfected CCLP1 xenografts mouse model. (A) Analysis of tumour volume by Vevo LAZR‐X photoacoustic imaging. Tumour growth was weekly monitored with a dedicated in vivo imaging system until 31 days after s.c. injection of GD3S‐transfected CCLP1 cells; tumours were obtained in NOD/SCID mice (*n* = 10 per group, *p ≤ 0.05). (B) Representative dissected tumour samples. (C) Ultrasound images of representative subcutaneous tumour masses. CTR‐T, tumour derived from control transfected cells; GD3S‐T, tumour derived from GD3S‐transfected CCLP1 cells. (D) CK7, Ki67, GD3S and haematoxylin eosin co‐staining by immunohistochemical analysis. Representative stainings are shown below the histograms (**p ≤ 0.01, ***p ≤ 0.001; Mann–Whitney U test GD3S‐T vs. CTR‐T). (E) Heatmap of different tumour samples based on qRT‐PCR arrays of statistically significant differential expressed genes focused on CSC pathways (84 genes). Unsupervised hierarchical clustering of genes using Euclidean distance as the similarity metric and complete linkage as the linkage method. Modified Z‐scores for individual genes, calculated as median‐centred log2 intensity values divided by the standard deviation, are shown using a blue‐to‐red gradient.

We next focused on the possible in vivo role of GD3S overexpression. To achieve this, we assessed tumour development subsequent to subcutaneous injection of CCLP1 overexpressing GD3S in NOD/SCID mice. In line with their enhanced in vitro sphere‐forming capability, cells transfected with GD3S exhibited heightened tumorigenicity, resulting in accelerated tumour growth compared to control cells. Tumours derived from GD3S‐transfected cells displayed volumes twice as large as those originating from control cells after 1 month (Figure 7A–C). Moreover, GD3S‐derived tumours (GD3S‐T) had a significantly higher volume compared to CTR‐T.

At the end of the treatment, tumour characterisation was performed. GD3S‐T tissues exhibited a higher number of Ki67‐positive cells, indicating increased proliferation as well as elevated levels of the biliary marker, CK7, and, as expected, GD3S (Figure 7D, Figure S8A). From a molecular standpoint, GD3S‐T tissues expressed a set of genes associated with stemness, including genes involved in self‐renewal (DNMT1, FGFR2), cell migration and metastasis (AXL, ZEB1, VIM, β‐catenin) and signal transduction related to Notch signalling (DLL4, MAML1), as well as AKT and PI3K/mTOR signalling (GSK3B). Moreover, genes related to cancer therapeutic targets, such as ABCG2, GSK3B, PTCH1, STAT3 and WEE1, were also upregulated (Figure 7E, Figure S8B).

### 2.3 Expression of GD3S Is Associated With Malignant Features in Patients With iCCA

**FIGURE 8:**
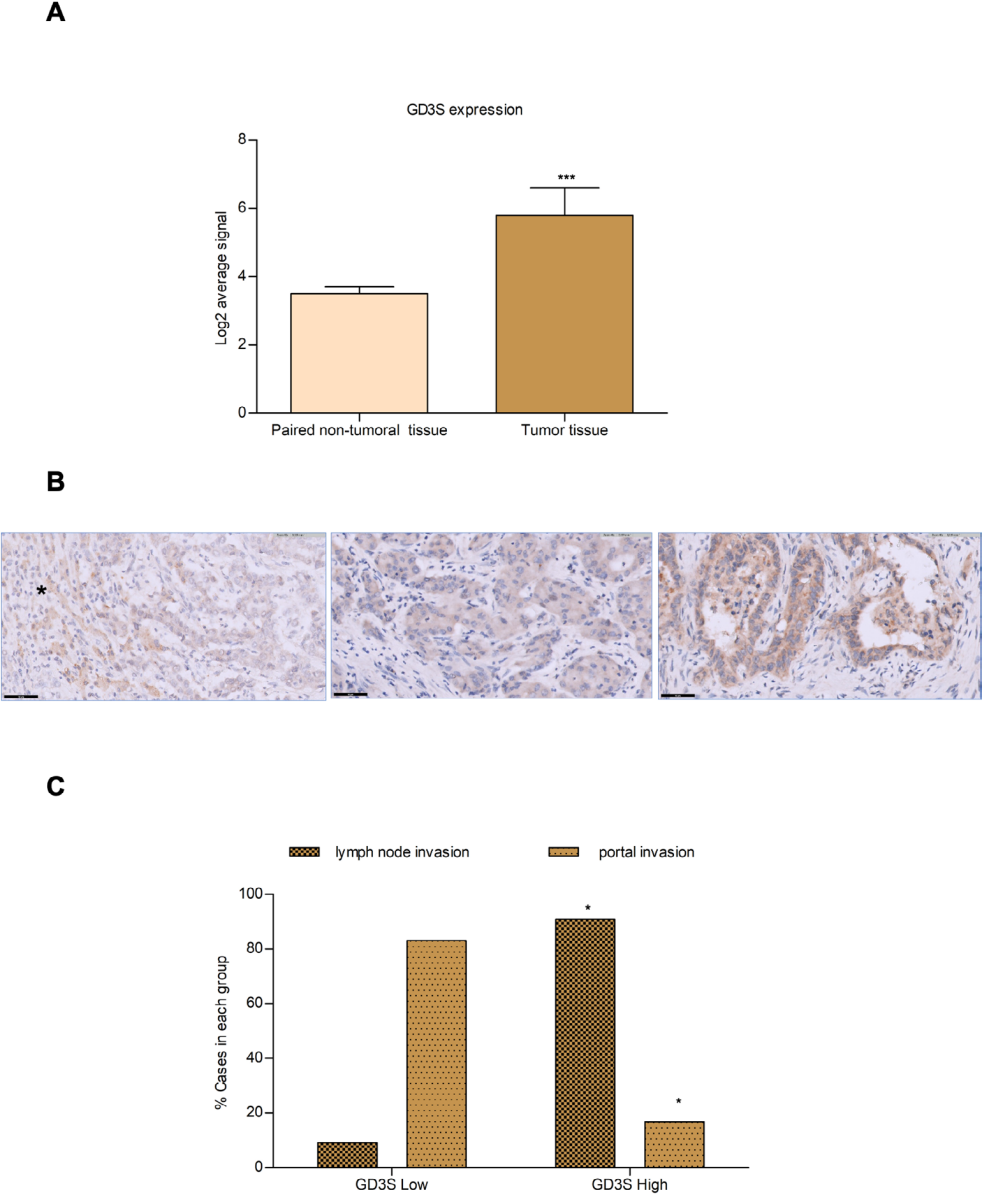
GD3S expression in human iCCA and clinical relevance. (A) Expression of GD3S evaluated in 104 tumour samples versus 59 matched surrounding non‐tumoral liver tissue of iCCA patients [17]; (B) GD3S expression in paraffin‐embedded sections of iCCA. Neoplastic cells can show faint (left), moderate (centre) or strong (right) immunoreactivity; the surrounding liver parenchyma is usually characterised by a low expression of GD3S (*), bar: 50 μm, 40× magnification (area: 0.119 mm^2^); (C) clinical relevance of GD3S expression in iCCA. The median expression of GD3S in a validating cohort of 39 iCCA cases [18] was used to define ‘Low’ and ‘High’ expressing groups. Statistical analysis of clinical data demonstrated a significant association of GD3S expression with portal invasion (*p* = 0.029), lymph node invasion (*p* = 0.03). Statistical analysis was performed using a two‐sided Fisher's exact test.

To investigate the involvement of ganglioside GD2 in the maintenance of a malignant phenotype in the human setting, we investigated the mRNA expression of GD3S in a published data set of iCCA patients (*n* = 104) (GSE26566) [17]. GD3S was upregulated in the tumour tissue compared to non‐tumoral tissue (Figure 8A).

However, further analysis of the potential prognostic significance of GD3S expression in publicly available cohorts (EGA00001000950, GSE26566) revealed no statistically significant correlation with overall survival (Figure S9).

In addition, the expression of GD3S was detected by immunohistochemistry in paraffin‐embedded specimens of iCCA tumours (Figure 8B), where iCCA cells specifically showed evident immunoreactivity for GD3S. Moreover, assessing the clinical relevance of GD3S expression in a validation iCCA cohort (*n* = 39) [18], a significant positive association with GD3S expression was found in cases presenting lymph node invasion, but with limited portal invasion (Figure 8C). In the same data set, GM2/GD2S expression was also higher in tumour samples (Figure S10A) and correlated with the presence of satellite nodules, lymph node invasion and recurrence (Figure S10B). However, as mentioned above, the link between GM2/GD2S and GD2 was less specific because it is related to the synthesis of both GM2 and GD2.

## 3 Discussion

Gangliosides, a complex family of glycosphingolipids, are increasingly recognised for their multifaceted roles in cancer progression, particularly in regulating invasion and stemness. The biosynthesis and degradation of gangliosides are tightly regulated by a network of enzymes, and dysregulation of these enzymes can significantly impact tumour behaviour. This intricate interplay between ganglioside metabolism and gene expression creates a complex network that governs cancer cell invasion and stemness.

Several surface molecules belonging to the glycosphingolipid family are typical of embryonic, pluripotent or multipotent stem cells [19, 20, 21]. In recent years, the involvement of complex ganglioside subclasses including the ganglioside GD2 has been reported in several types of tumours, such as glioblastoma [22], sarcomas [23], bladder [24], breast [8, 25, 26, 27] and prostate cancer [28] and melanoma [14]. Moreover, CSC, a subpopulation with specific biological features within the tumour mass [29], often show sustained GS synthesis [19, 20, 21] and, in particular, GD2 [8, 24, 26]. Despite the potential relevance of the GS system in oncology and specifically in CSC biology, no data in human iCCA or its stem‐like subsets are available. The data reported herein provide a detailed characterisation of the GS profile in iCCA cells and of the functional role of GD2, a complex ganglioside, in the biology of iCCA CSC. Comparing two well‐established cell lines, HUCCT1 and CCLP1, an interesting cell line‐specific GS profile was observed. CCLP1 showed high levels of the GM2 and GM1, monosialo‐GS, whereas GM3 was more abundant in HUCCT1. Of note, when iCCA cells were cultured as spheres, a system which leads to culture enrichment with CSC, the GS pattern was modified in the same direction in the two cell lines. GD2 increased in both HUCCT1 and CCLP1 spheres and was significantly more abundant on the external layer of the plasma membrane. Based on these results, we analysed the expression of GD3S and GM2/GD2S, specific synthases operating in GD2 formation. GD3S catalyses the transfer of a sialic acid residue onto GM3 to synthesise GD3, while the glycosyltransferase GM2/GD2S adds N‐acetylgalactosamine residues to generate GD2. Thus, the amount of GD2 indirectly depends on GD3S, which was found to be expressed at high levels in the SPH of both iCCA lines. These data identify GD3S as a critical enzymatic machinery leading to accumulation of complex GS such GD2 in iCCA SPH.

iCCA SPH expressing GD2 on the membrane had higher expression of genes associated with pluripotency, stemness and drug resistance. Gain‐of‐function experiments in cells overexpressing GD3 allowed us to establish the functional relevance of the GD3S/GD2 axis in iCCA cells. As expected, these cells had a higher content of GD2 and acquired several features of aggressive cancer, such as higher resistance to drugs used in iCCA chemotherapy and enhanced ability to invade a basement membrane‐like matrix. Intriguingly, the ability of GDS3‐overexpressing cells to generate spheres was significantly increased compared to cells transfected with the control vector, reinforcing the functional link between CSC and GD2. This observation is supported, at least in part, by the results obtained with PMPP, a specific GS biosynthetic inhibitor, on sphere‐forming ability and expression of CSC‐related genes. Although this approach is not entirely specific to the GS3/GD2 pathway, it suggests that interference with the ganglioside pathway has the potential to be translated to the clinical setting once effective and specific pharmacological compounds become available.

GD3S and/or GD2 are the focus of current investigation for their role in cancer promotion and cancer stemness [13, 15, 16, 28]. Ganglioside GD2 plays a broad spectrum of functional roles [30, 31, 32]. In humans, GD2 is physiologically expressed in the central nervous system, affecting the composition and formation of membrane microdomains involved in cell interactions, surface receptor signalling, and the adhesion process [33]; is also implicated in T‐cell dysfunction [34], and several other immunological activities. The expression of GD2 has been also found associated with other several physiological and pathological conditions, such as developmental processes [35], metabolic stress [26], enhancement of cell survival and invasion [9, 35]. Frequently, studies on GD2 and GD3S have been performed jointly, due to strict connection in the synthesis pathway, and have highlighted several roles potentially relevant in cancer, such as maintenance of a microenvironment quiescent state or regulation of DNA‐methylation [16, 36, 37].

Several pathways connecting ganglioside metabolism to invasion, including receptor tyrosine kinase signalling, such as EGFR and PDGFR, which are frequently implicated in cancer invasion [8], have been reported. These receptors activate downstream signalling cascades, including the MAPK and PI3K/Akt pathways, ultimately promoting EMT and enhancing cell motility. Moreover, gangliosides influence integrin‐mediated cell adhesion and signalling, affecting how cancer cells interact with the extracellular matrix [38]. This interaction is crucial for cell migration and invasion, as it allows tumour cells to detach from the primary tumour mass and invade surrounding tissues. Moreover, the expression and activity of matrix metalloproteinases (MMPs), enzymes responsible for ECM degradation, are also influenced by ganglioside metabolism. By regulating MMP activity, gangliosides contribute to the breakdown of the ECM, facilitating tumour cell invasion and metastasis.

Furthermore, specific gangliosides, such as GD2 and GD3, have been identified as markers of cancer stem cells and are essential for maintaining their self‐renewal capacity [39]. These gangliosides modulate key stemness‐associated signalling pathways, including Wnt and Notch, which are crucial for preserving the undifferentiated state and tumour‐initiating potential of CSCs.

Ganglioside GD2 may modulate tumour signalling through lipid raft complexes, membrane microdomains enriched in gangliosides, serve as critical platforms for signal transduction. Their activation is largely dependent on interactions with gangliosides.

In the present study, transcriptomic analysis showed that higher expression of GD2 was associated with enhanced activity of relevant pathways, such as development, morphogenesis, MAPK signalling and EMT. Moreover, this transcriptomic approach allowed us to identify factors, such as epiregulin and ephrin B2, that will deserve specific evaluation in further studies to assess their potential relevance in the context of iCCA biology.

To provide evidence that GD2 synthesis confers to iCCA cells a more severe malignant phenotype, GD3S‐overexpressing cells were used to generate tumour xenografts in NOD/SCID mice. These experiments were conducted in CCLP1 cells, where higher membrane GD2 levels were associated with a non‐altered GS profile and showed that tumours grew more rapidly, reaching a volume 2–3 fold greater than in control mice. Thus, the in vitro results indicating acquisition of a more invasive potential upon enrichment with GD2 could be reproduced in a mouse model in vivo. To expand the translational potential of this study, we also analysed available databases of patients with iCCA. The regulatory enzyme, GD3S, was expressed at higher levels in tumour tissue than in neighbouring liver.

At a functional level, GD3S expression was associated with lymph node invasion, an observation well‐fitting with the increase invasive potential observed in the in vitro studies and the transcriptomic data indicating enrichment with pathways related to cell motility in cells overexpressing GD3S. The GM2/GD2S was also found to be strongly expressed in both iCCA‐SPH and in tumour samples from the patient data set. GM2/GD2S expression is closely correlated with the presence of satellite nodules, lymph node invasion and recurrence, although its link with GD2 is less stringent because this enzyme leads to the synthesis of both GM2 and GD2. These data open the way to future investigation aimed at detailing the regulation of the specific transferases in GS synthesis and their kinetics.

Additionally, the respective relevance of the pathways responsible for mediating the pro‐malignant effect of GD2 warrants additional experimentation with genetic or pharmacologic approaches. Along these lines, the unavailability of specific inhibitors for each step of GS synthesis represents an obvious limitation and the use of PPMP, the effects of which were reported herein, needs to be extended with other compounds.

In conclusion, these results indicate that the GS composition is modulated in iCCA and highlight a novel role of the synergy obtained by merging GD3S expression with ganglioside GD2 as a regulator of the biology of iCCA and of cancer stemness in this tumour. Efforts should be put into studies on the GS pathway to examine in depth, identifying signal pathways useful for the management of this deadly cancer.

## 4 Materials and Methods

### 4.1 Colture Conditions

CCLP1 and HUCCT1 cells, from intrahepatic bile duct cancer tissue, were a kind gift from Dr. AJ Demetris, University of Pittsburgh. Cell lines were cultured as described [3, 4].

### 4.2 Sphere Generation

The iCCA cells were grown in anchoring‐independent conditions into poly 2‐hydroxyethyl methacrylate (poly‐HEMA)‐coated dishes (Sigma Aldrich) with selective serum‐free DMEM/F12 medium supplemented with 1X B27 supplement without vitamin A (Life Technologies), 20 ng/mL EGF, and 20 ng/mL bFGF (R&D Systems) [3, 4]. To determine SPH‐forming ability, 500 iCCA cells/well in a 96 multiwell were grown in anchoring‐independent conditions with selective serum‐free medium. After 7 days, pictures were taken to measure the number of iCCA‐SPH using a Leica DMi1microscope (Leica). Average number of formed spheres microscopic field (20×) over five fields.

### 4.3 iCCA Cell Transfection With GD3S

For the transfection of GD3S in HUCCT1 and CCLP1 cells, ST8SIA1 Lentiviral Vector (Human) (CMV) (pLenti‐GIII‐CMV, LV701751) (#45655062, Applied Biological Materials Inc.) and pLenti‐III‐Blank Vector (LV587, as a blank control, Applied Biological Materials Inc) (https://www.abmgood.com/ST8SIA1‐Lentiviral‐Vector‐Human‐CMV‐pLenti‐GIII‐CMV‐45655062.html) (Scheme S2). Transfection was performed according to Applied Biological Materials Inc. instructions, and after lentivirus infection, iCCA‐infected cells were selected by puromycin (1 ug/mL).

### 4.4 D‐*Threo*‐1‐Phenyl‐2‐Palmitoylamino‐3‐Morpholino‐1‐Propanol (PPMP) Treatment

To test the biosynthetic GS‐pathway, iCCA cells (MON and SPH) were treated with PPMP (#870792P‐5MG, Merck), a specific inhibitor of glucosylceramide synthase (first step of GS synthesis). PPMP was dissolved in CH_3_OH:H_2_O (19:1) at a concentration of 0.5 mg/mL, dried under N_2_ flow, at 37°C and resuspended in a solution of BSA in 4 mg/mL of BSA in H_2_O. This solution was diluted in a culture medium at the final concentration of 2.5 μM. Untreated cells were used as a control and incubated under the same experimental conditions without PPMP.

### 4.5 Lipid Analysis

The iCCA cells (‐MON, ‐SPH or GD3S‐transfected cells) were fed with [1‐^3^H]‐sphingosine to metabolically label cell sphingolipids at the steady state as previously described [40]. Briefly, [1‐^3^H]‐sphingosine was solubilised in a complete culture medium at the final concentration of 36 nM (specific radioactivity 1.06 Ci/mmol) and administered to the cells. After 2 h of incubation, the medium was substituted with a fresh culture medium without radioactive sphingosine for 48 h after which PPMP was administered for 72 h. At the end of PPMP treatment, cells were harvested and lysed with H_2_O supplemented with proteinase. Protein concentration was assessed using the DC protein assay kit according to manufacturer's instructions. The cell lysates were then lyophilised and total lipids were extracted with CHCl_3_:CH_3_OH:H_2_0 (2:1:0.1, v:v:v) and separated from the pellet by centrifugation at 13 000×*g* for 15 min, followed by a second extraction with CHCl_3_:CH_3_OH (2:1, v:v). Total lipid extracts (TLE) were subjected to a two‐phase partitioning by adding 20% water and centrifuging at 13 000×*g* for 15 min, which resulted in the separation of an aqueous phase (AP) containing gangliosides and an organic phase (OP) containing the other lipids. The radioactivity associated with TLE, AP and OP was determined by liquid scintillation counting by beta‐counter (PerkinElmer). Lipids of the AP were resolved by bi‐dimensional HPTLC using for the first run the solvent system 50:42:11 CHCl_3_:CH_3_OH:0.2% aqueous CaCl_2_ (v:v:v) and for the second run 10:75: 15 CH_3_CN: CH_3_CHOHCH_3_: aqueous KCl 50 mM (v:v:v). Radioactive lipids were detected by digital autoradiography (Beta‐Imager TRacer Betaimager, BioSpace Laboratory, Paris, France), identified by comigration with authentic standards, and quantified using the M3 Vision software (BioSpace Laboratory, Paris, France).

### 4.6 Flow Cytometry

To analyse the expression of the ganglioside GD2 on the cell surface, 1 × 10^6^ cells were incubated with primary antibody anti‐human‐Disialoganglioside GD2 (Purified 14.G2a Hu 0.1 mg, NR, Nannini srl, Italy) for 30 min/1 h at 4°C and then with Alexa Fluor 488‐conjugated secondary antibody (#A11017, Life Technologies Italia) for 30 min at 4°C. Expression of GD2 was measured at sixth day of cell growth with a FACSCanto flow cytometer (Becton–Dickinson, Franklin Lakes, NJ, USA). Background signal was determined using matched isotype control. 7AAD (#559925, BD Biosciences, USA)‐negative cells were considered for analysis.

For cell sorting experiments, 10 × 10^6^ cells were incubated with primary antibody anti‐human‐GD2 (Purified 14.G2a Hu 0.1 mg, NR, Nannini srl, Italy), PE‐conjugated CD133 (#372804, Biolegend, USA), APC Fire Epcam (#324234, Biolegend, USA) for 30 min at 4°C. Afterwards, cells were stained with fluorochrome‐conjugated secondary antibody; Alexa Fluor 488‐conjugated secondary antibody (#A11017, Life Technologies, Italy) for 30 min at 4°C. Expression of CD133 and EpCAM was measured in GD2‐ and GD2+ sphere cells separated using BD FACSMelody Cell Sorter (BD). Background signal was determined using matched isotype control. 7AAD (#559925, BD Biosciences, USA)‐negative cells were considered for analysis.

### 4.7 Quantitative Real‐Time Polymerase Chain Reaction (RT‐PCR)

For in vitro study, total RNA was extracted with the RNeasy kit (Qiagen) according to the manufacturer's instructions. The RNA concentration and quality were determined using an optical NanoDrop ND1000 spectrophotometer (ThermoFisher Scientific). Total RNA (1 μg) was retro‐transcribed with a High‐Capacity cDNA Reverse Transcription Kit (Applied Biosystems). Relative gene expression was calculated using 2‐ΔCt method. The mRNA levels of Actin were used for normalisation. Sequences of used primers are listed in Table S1 [41].

In addition, total RNA (3 μg) from in vivo mouse tumours was reverse transcribed using QuantiNova Reverse Transcription kit (Qiagen). RT‐PCR was performed using QuantiNova LNA PCR Focus Panel Human Cancer Stem Cells 96‐well plates (PAHS‐176ZD, Qiagen). In each 96‐well plate, a tumour sample for each group were tested for 84 genes specifically associated with liver cancer pathways. The expression values were calculated with the ΔΔCt method, using the average of ACTB, GAPDH, RPLP0 and HPRT1 as housekeeping genes as reference. A cut‐off of at least 1.5‐fold increases and 0.5‐fold decreases were considered significant. Moreover, the same tumour samples for each group were tested for additionally genes associated with stemness features (CSC, EMT and drug transporters; Table S1). Total RNA (1 μg) from in vivo mouse tumours was retro transcribed with a High‐Capacity cDNA Reverse Transcription Kit (Applied Biosystems). Relative gene expression was calculated using 2‐ΔΔCt method. The mRNA levels of Actin were used for normalisation. Data are mean ± SEM (*n* = 3). A cut‐off of at least 1.5‐fold increases and 0.5‐fold decreases were considered significant.

Afterwards, heatmap representation was performed by MeV version 4.9.0 (Saeed et al., 2006) using an unsupervised hierarchical clustering, with Euclidean distance as the similarity metric and complete linkage as the linkage method, which links clusters based on the maximum distance between their members.

### 4.8 Western Blot Analysis

Cells were lysed at 4°C with lysis buffer (1% Triton X‐100, 50 mmol/L Tris–HCl, pH 7.4, 150 mmol/L NaCl, 1 mmol/L EDTA, 1 mmol/L sodium orthovanadate, 2 mmol/L PMSF and 1 mmol/L each of leupeptin and pepstatin). After 30 min of lysis, cellular extracts were centrifuged for 20 min at 14000 rpm, and the supernatant was used for Western blot experiments as detailed elsewhere [4]. Antibodies were used according to the manufacturer's instructions. Immunoblots were incubated overnight at 4°C with primary antibody in 1% BSA in PBS. ST8SIA1 primary antibody (Rabbit Polyclonal antibody catalogue # 24918‐1‐AP, Proteintech, www.ptglab.com) was used. Immunoblot were then incubated with secondary antibody α‐rabbit/mouse (1:4000) in 1% BSA in 1× DPBS for 30 min, then with Horseradish peroxidase (HRP)‐conjugated tertiary antibody α‐rabbit/mouse for 15 min monoclonal anti‐vinculin antibody produced in mouse (V9131, Sigma) were used as an internal control (1:1000). Quantification of the signal was obtained by chemiluminescence detection on an Image Quant Las4000 (GE Healthcare Life Sciences) and subsequent analysis conducted with ImageJ software.

Quantification of the signal was obtained by chemiluminescence detection on an Image Quant Las4000 (GE Healthcare Life Sciences, Little Chalfont, UK) and subsequent analysis with ImageJ software.

### 4.9 Migration Assay

Migration was measured in Boyden chamber equipped with 8 μm pore filters (Millipore Corp) coated with rat tail collagen (20 μg/mL) (Collaborative Biomedical Products, Bedford, UK), as described in detail elsewhere [3]. After 6 h incubation at 37°C, iCCA‐transfected cells migrated to the underside of the filters were fixed, stained with Diff Quick, mounted and counted at 40× magnification. The values for migration were expressed as the average number of migrating cells per microscopic field over five fields. Each experiment was performed in triplicate.

### 4.10 Cell Survival Assay

A total of 8 × 10^4^ iCCA‐transfected cells were seeded in 96 multiwell plates. Then cells were treated with five fluorouracil, oxaliplatin and cisplatin, as described in detail elsewhere [3]. Medium was removed and a 0.5% crystal violet solution in 20% methanol was added. After 5 min of staining, the fixed cells were washed with phosphate‐buffered saline (PBS) and solubilised with 100 μL/well of 0.1 M sodium citrate, pH 4.2. The absorbance at 595 nm was evaluated using a microplate reader (HiPo biosan, Bio Class).

### 4.11 Confocal Microscopy

Sections of OCT‐embedded spheroids of human CCLP1 and HUCCT1 cells (8 μm thick) were shaked in PBS for 30 min to remove OCT, unmasked in 1x sodium citrate buffer at 98°C for 10 min and permeabilized in PBS with 0.1% Triton X‐100 for 5 min. The non‐specific antibody binding sites were blocked by applying a solution of 10% normal goat serum (NGS; Sigma‐Aldrich) in PBS for 1 h at room temperature (RT). Then, the frozen sections were incubated overnight at 4°C with mouse anti‐human Disialoganglioside GD2 (1:100, BD Biosciences, San Jose, CA, USA). The immunoreactions were revealed by incubation with specific goat anti‐mouse Alexa Fluor 488‐conjugated IgG (1:200; Molecular Probes Inc., Eugene, OR) for 1 h at RT. Counterstaining were performed with Wheat Germ Agglutinin (WGA) conjugated with Alexa 555 (1:100 for 10 min, Molecular Probes) to reveal cell plasmamembrane and with 4′,6‐diamidino‐2‐phenylindole (DAPI; Chemicon International, Temecula, CA, USA; 1:1000 for 10 min) for nuclei. Negative controls were performed by replacing the primary antibody with a non‐immune serum. After washing in PBS, the immunolabelled sections were mounted with a Fluoromount Aqueous Mounting Medium (Sigma) and observed with a Leica Stellaris 5 confocal laser scanning microscope (Leica, Manheimer, Germany) equipped with a Plan‐Apo 63×/1.4NA oil immersion objective. For each field, series of optical sections (1024 × 1024 pixels, 0.18 × 0.18 μm pixel size) at intervals of 0.3 μm, were obtained and superimposed to create a single composite image.

### 4.12 In Vivo Experiment

Animal experiments were performed in accordance with national guidelines and approved by the ethical committee of the Animal Welfare Office of the Italian Health Ministry. All procedures conformed to the legal mandates and the Italian guidelines for the care and maintenance of laboratory animals. All animals received human care and study protocols comply with the institution's guidelines. Studies involving animal experiments conform to the Animal Research: Reporting of In Vivo Experiments (ARRIVE) guidelines (http://www.nc3rs.org.uk/arriveguidelines), developed by the National Centre for the Replacement, Refinement and Reduction of Animals in Research (NC3Rs) to improve standards and reporting of animal research. Male NOD/SCID mice (*n* = 10 per group) of 6 weeks (Charles River Laboratories International) were subcutaneously injected with 5 × 10^6^ CCLP1 GD3S‐ and control transfected cells.

### 4.13 Ultrasound and Photoacoustic Imaging

Tumour volumes were determined in vivo imaging system (Vevo LAZR‐X photoacoustic imaging). High‐resolution ultrasound (US) imaging was done by performing 3D acquisition in B‐Mode on live mice and by using VevoLAZR‐X imaging station (Fujifilm Visualsonics). The acquisitions to evaluate the tumour growth were performed weekly, starting from the day before the first treatment, until the experimental endpoint. During the analysis, mice were anaesthetised with a continuous flow of isoflurane (initial induction at 4% and maintenance at 2%) and placed on a mouse handling table, heated at 37°C, in a prone position. ECG, respiration rate and body temperature were monitored during the analysis. The 55‐MHz transducer was used for echography. Data obtained were analysed using Vevo LAB software (Fujifilm Visualsonics) to measure the tumour volumes.

### 4.14 Imunohistochemical Analysis of GD3S Derived Tumours

Tumours from mouse were collected, fixed in 4% neutral‐buffered formalin for 24 h and embedded in paraffin wax. Sections of 5 μm were used to perform Haematoxylin/Eosin (H&E) and IHC analysis. Slides were stained with following antibody:anti‐human ki‐67(Dako Omnis ready to use), anti‐cytokeratin7 (#181598 Abcam, 1:1000), anti‐ST8SIA1 (#24918‐1‐AP proteintech, 1:100). Immunohistochemistry was performed using the Leica BOND‐MAX automated system (Leica Microsystems). Slides were developed with 3′3‐diaminobenzidine (DAB) and counterstained with haematoxylin. The antibody staining intensity for cytokeratin7 was associated with an intensity histoscore (H score) of 0, +1, +2, +3, evaluated by at least two researchers. Staining quantification of ki‐67 was performed using ImageJ. Images were obtained with a slide scanner (Aperio LV1, Leica Biosystems) and analysed with ImageScope Software.

### 4.15 Transcriptomic Analyses in iCCA and GD3S‐Transfected Cells

For gene expression profiling, RNA quality and quantity were assessed using Agilent 2100 bioanalyzer (Agilent Technologies) and NanoDrop ND‐1000 spectrophotometer (Thermo Fisher Scientific, Waltham, Massachusetts, USA), respectively. Gene expression profiling was carried out using the one‐colour labelling method by means of Low Input Quick Amp Labeling Kit (Agilent Technologies): labelling, hybridisation, slide washing and scanning were performed following the manufacturer's protocols. Briefly, mRNA from 100 ng of totRNA was amplified, labelled with Cy3 and hybridised on Agilent Human Gene Expression v3 8 × 60 K microarrays. Slides were then scanned using the Agilent Scanner version C (G2505C, Agilent Technologies).

The LIMMA (Linear Models for Microarray Analysis) package was used to analyse gene expression profiles of GD3S‐transfected and control HUCCT1 and CCLP1 cell lines. Raw intensity values were background subtracted (method = normexp, offset = 50) and normalised using the quantile for the between array normalisation. Duplicated probes were averaged. Over‐represented biological processes, molecular functions and cellular components of the Gene Ontology (GO) were investigated with the functional annotation tool available within DAVID v.2022q3 (https://david.ncifcrf.gov/). MetaCore (Clarivate Analytics, Philadelphia, PA, USA) was used for network and pathway analysis.

GSEA v.4.3.2 (https://www.gsea‐msigdb.org/gsea/index.jsp) was used to evaluate significant enrichment in predefined sets of genes.

Raw and processed transcriptomic data were deposited on the GEO Omnibus database GSE238179.

### 4.16 GD3S Expression in Human iCCA and Clinical Relevance

Profiles of gene expression. Gene expression profiles of 104 CCA patients and 58 matched, histologically normal and tumour‐adjacent liver tissues [17] were obtained from GEO (GSE 26566). Average signals were tested for normality distribution, plotted and pairwise comparison was performed using Mann–Whitney test (*p < 0.05, **p < 0.01, ***p < 0.001, ****p < 0.0001). For The Cancer Genome Atlas (TCGA, ref) gene expression data were obtained from Firebrowse (Broad Institute). Data were tested for normality distribution, plotted and pairwise comparison was performed using unpaired t‐test (*p < 0.05, **p < 0.01, ***p < 0.001, ****p < 0.0001). Correlation of GD3S expression with survival was performed by stratifying CCA patients (EGA00001000950, GSE26566) by median expression of GD3S and generation of Kaplan–Meier curves. Survival curves were compared with the log‐rank test (Prism 9.4.1).

### 4.17 Immunohistochemical Analysis of GD3S Protein Level in Human iCCA Tissues

A total of 57 formalin‐fixed, paraffin‐embedded human liver tissue specimens were analysed for the immunohistochemical expression of GD3S. All specimens were obtained after surgical resection and collected in the tissue bank at Humanitas Clinical Institute (Rozzano, Italy) in accordance with informed consent retrieved from patients and local ethics committee approval conforms to the ethical guidelines of the 1975 Declaration of Helsinki as reflected in a priori approval by the institution's human research committee.

The immunohistochemical expression of GD3S (#24918‐1‐AP‐20UL Polyclonal Antibody, DBA) was performed on the automated Leica Microsystems Bondmax (Leica). Both cytoplasmic and nuclear staining were retained for scoring. Immunostaining was semi‐quantified using a three‐tier scoring based on the intensity of GD3S. Regarding the staining intensity, 0 = negative, 1 = weak, 2 = moderate, 3 = strong.

### 4.18 Clinical Relevance of GD3S Expression in iCCA Patients

The median expression of GD3S in a validating cohort of 39 iCCA cases [18] was used to define ‘Low’ and ‘High’ expressing groups. Statistical analysis was performed using a two‐sided Fisher's exact test.

### 4.19 Statistical Analysis

Student *t*‐test was used for pairwise comparison. Statistical analysis was performed by GraphPad version 5 (GraphPad Prisme Software Inc., La Jolla, CA) or as specified in the previous paragraphs, *p* < 0.05 were considered statistically significant.

## Author Contributions

A.M. and C.R. designed the study and wrote the manuscript. A.M., M.P., A.G., M.C., E.S.L. T.L., B.P., I.T., E.R., C.C., J.B.A., M.L., C.C., V.L.B., B.Y., M.A., E.V.C., C.P.N., P.O., A.T., D.N., A.V., L.M., L.D.T., G.C., S.M., A.A., F.M. and C.R. provided materials, performed the experiments, collected the data, and analysed the results. C.R. and F.M. supervised the project and critically revised the manuscript. All authors have read and approved the final manuscript.

## Ethics Statement

The study was performed in accordance with the guidelines of the Helsinki Declaration.

## Consent

Informed consent was obtained from all patients.

## Conflicts of Interest

The authors declare no conflicts of interest.

## Permission to Reproduce Material From Other Sources

No materials reproduced from other sources in this manuscript.

## Supporting information


Data S1.

## Data Availability

The data that support the findings of this study are available on request from the corresponding author. The data are not publicly available due to privacy or ethical restrictions.
