# Supplementary material for: Ganglioside GD2 Contributes to a Stem‐Like Phenotype in Intrahepatic Cholangiocarcinoma
Source: Liver Int. 2024 Dec 26;45(1):e16208. doi: 10.1111/liv.16208 (PMC11684508; doi:10.1111/liv.16208)
Supplement: Supplementary file 1 — Data S1. [file LIV-45-0-s001.zip › REV_SupplementaryMaterial-Final_CLEAN.docx]

**SUPPLEMENTARY MATERIAL**

**Introduction**

**
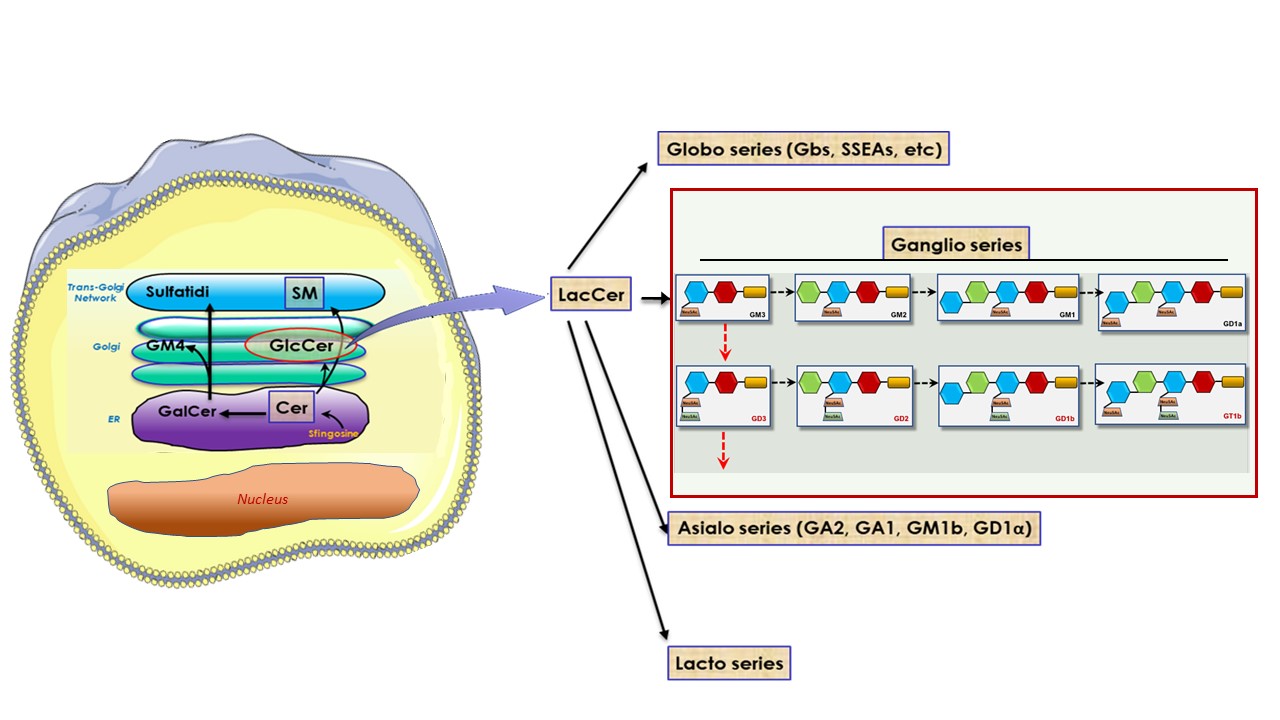
Scheme S1. Biosynthetic pathway of gangliosides, starting from glycosphingolipids.**

**Suppl. Figure Legends**

**Figure S1. Stem-like molecular features of 3D sphere culture.** CCLP1 and HUCCT1 cells were grown as monolayer (MON) or as spheres (SPH), then RNA was extracted. Expression of different stem-like genes is reported as 2^deltaCT. Mean ± SEM (n=4, *p≤0.05, ** p≤0.01, *** p≤0.001 SPH vs MON).

**Figure S2.** **Expression of GM2/GD2 synthase in iCCA-MON and -SPH.** The GM2/GD2 synthase was evaluated in MON and SPH of iCCA cells (HUCCT1 and CCLP1) and reported as fold expression relative to MON (n=5, **p<0.01 SPH vs MON).

**Figure S3.** **Molecular characterization of GD2^pos^ cells in CCLP1-SPH**. FACS-sorted GD2^pos^ and GD2^neg^ CCLP1-SPH cells were evaluated for expression of different genes involved in CSCs pathways, EMT and drug resistance expressed as fold changes normalized to mean expression of GD2^neg^ cells. Mean ± SEM (n=3, **p≤0.01, ***p≤0.001, GD2^pos^ vs GD2^neg^). GD2^pos^, GD2 positive cells; GD2^neg^, GD2 negative cells.

**Figure S4. GS features in iCCA cells overexpressing GD3S.** (A) GD3S gene expression levels following transfection presented as 2^deltaCT. Mean ± SEM (n=5, ***p ≤0.0001 GD3S-stably transfected vs CTR). (B) FACS profiles of HUCCT1 and CCLP1 GD3S-stably transfected. Representative dot plots with percent of GD2^pos^ determined by FACS in iCCA cells overexpressing GD3S**.**

**Figure S5.** **Genes related to morphogenesis/development.** Specific list of genes associated to morphogenesis / developmental processes identified by gene ontology analysis. In red genes common to both iCCA cell lines.

**Figure S6. Common pathways associated to morphogenesis identified by gene ontology analysis.** All the processes listed are significant (p < 0.05).

**Figure S7. Common pathways associated to developmental process identified by gene ontology analysis.** All the processes listed are significant (p < 0.05).

**Figure S8. Molecular characterization of tumor derived from GD3S-transfected CCLP1 cells.** (A) Evaluation of the GD3 synthase in tumor derived from GD3S-transfected CCLP1 cells. The mRNA expression level was reported as 2^deltaCT. Data are Mean ± SEM (n=5, ***p≤0.001 GD3S-T vs CTR-T). (B) Unsupervised hierarchical clustering of CSC genes in GD3S+ vs control in vivo tumors, using Euclidean distance as the similarity metric and complete linkage as the linkage method. Modified Z-scores for individual genes, calculated as median-centered log2 intensity values divided by the standard deviation, are shown using a blue-to-red gradient.

**Figure S9.** **Expression of GD3S and survival in iCCA patients.** Kaplan-Meyer plot showing overall survival in iCCA patients stratified according to good or bad prognosis in a public dataset (EGA00001000950, GSE26566).

**Figure S10. Expression of GM2/GD2 synthase profile from surgical resected CCA samples and clinical relevance.** (A) The GM2/GD2 synthase was evaluated in tumor tissue compared to that in paired non-tumoral tissue (reported as log2 Avg); (B) Clinical relevance of GM2/GD2 synthase (Khi2 test, High vs Low).

**Materials and Methods**

**Scheme S2.** Vector Details. ST8SIA1 Lentiviral Vector (Human) (CMV) (pLenti-GIII-CMV)

(<https://www.abmgood.com/ST8SIA1-Lentiviral-Vector-Human-CMV-pLenti-GIII-CMV-45655062.html>)


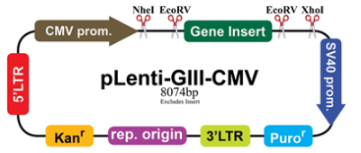


**Table S1**

**Index list of primer sequences used for RT-qPCR analysis**

Gene name Forward sequence (5ʹ→3ʹ) Reverse sequence (5ʹ→3ʹ)

| **Gene** | **Primer Forward** | **Primer Reverse** |
| --- | --- | --- |
| **GD3S** | GCGATGCAATCTCCCTCCT | TTGCCGAATTATGCTGGGAT |
| **GM3S** | ATCGGTGTCATTGCCGTTGT | TTCATAGCAGCCATGCATTGA |
| **GM2/GD2S** | CAGCGCTCTAGTCACGATTGC | CCACGGTAACCGTTGGGTAG |
| **CD13** | CAGTGACACGACGATTCTCC | CCTGTTTCCTCGTTGTCCTT |
| **CD24** | TAGGTACCACTATGGGCAGAGCAATGG | CCGGAATTCCGTTAAGAGTAGAGATGC |
| **CD44** | GTGATGGCACCCGCTATG | ACTGTCTTCGTCTGGGATGG |
| **CD133** | GCTTCAGGAGTTTCATGTTGG | GGGGAATGCCTACATCTGG |
| **c-MYC** | CGGAACTCTTGTGCGTAAGG | ACTCAGCCAAGGTTGTGAGG |
| **NANOG** | GTCTCGTATTTGCTGCATCG | GAAACACTCGGTGAAATCAGG |
| **NFκB1** | CTCCGAGACTTTCGAGGAAATAC | GCCATTGAAGTTGGTAGCCTTCA |
| **BMI1** | TTGCTTTGGTCGAACTTGG | GTGCTTCTTTTGCAGACTGG |
| **BMP4** | AGCGTAGCCCTAAGCATCAC | AGTCATTCCAGCCCACATCG |
| **KLF4** | AGACAGTCTGTTATGCACTGTGG | TGTTCTGCTTAAGGCATACTTGG |
| **OCT4** | TTGTGCCAGGGTTTTTGG | ACTTCACCTTCCCTCCAACC |
| **SOX-2** | ATGGGTTCGGTGGTCAAGT | GGAGGAAGAGGTAACCACAGG |
| **HNF4** | CTCGTCGACATGGACATGGCCGACTAC | GGCTTGCTAGATAACTTCCTGCTTGGT |
| **LIN28A** | CAAAAGGAAAGAGCATGCAGAA | ATGATCTAGACCTCCAGAGTTGTAGC |
| **GAPDH** | GATCATCAGCAATGCCTCCT | TGTGGTCATGAGTCCTCCCA |
