# Supplementary figures and images for: Ganglioside GD2 Contributes to a Stem‐Like Phenotype in Intrahepatic Cholangiocarcinoma
Source: Liver Int. 2024 Dec 26;45(1):e16208. doi: 10.1111/liv.16208 (PMC11684508; doi:10.1111/liv.16208)

## Slide 1
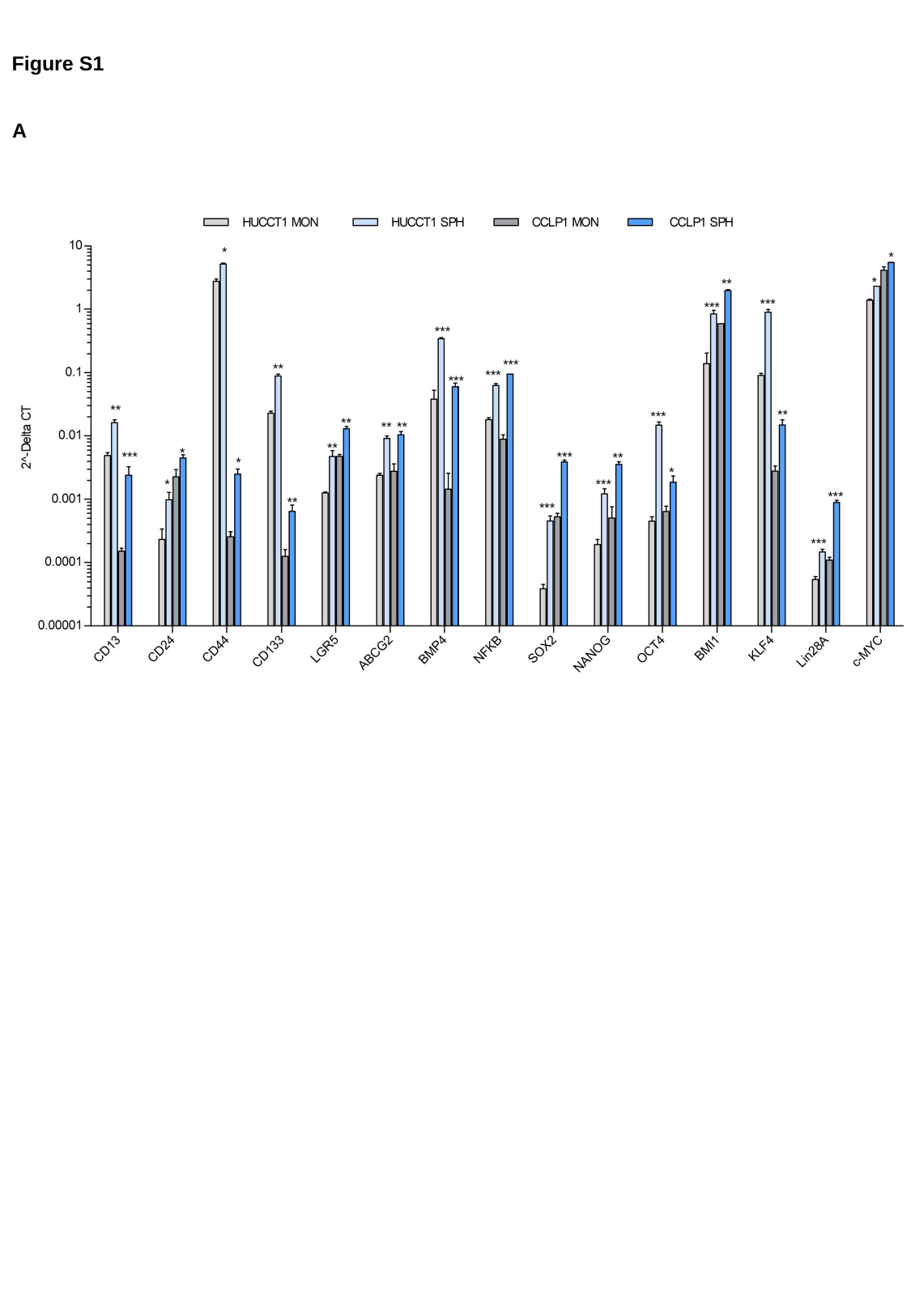

## Slide 2
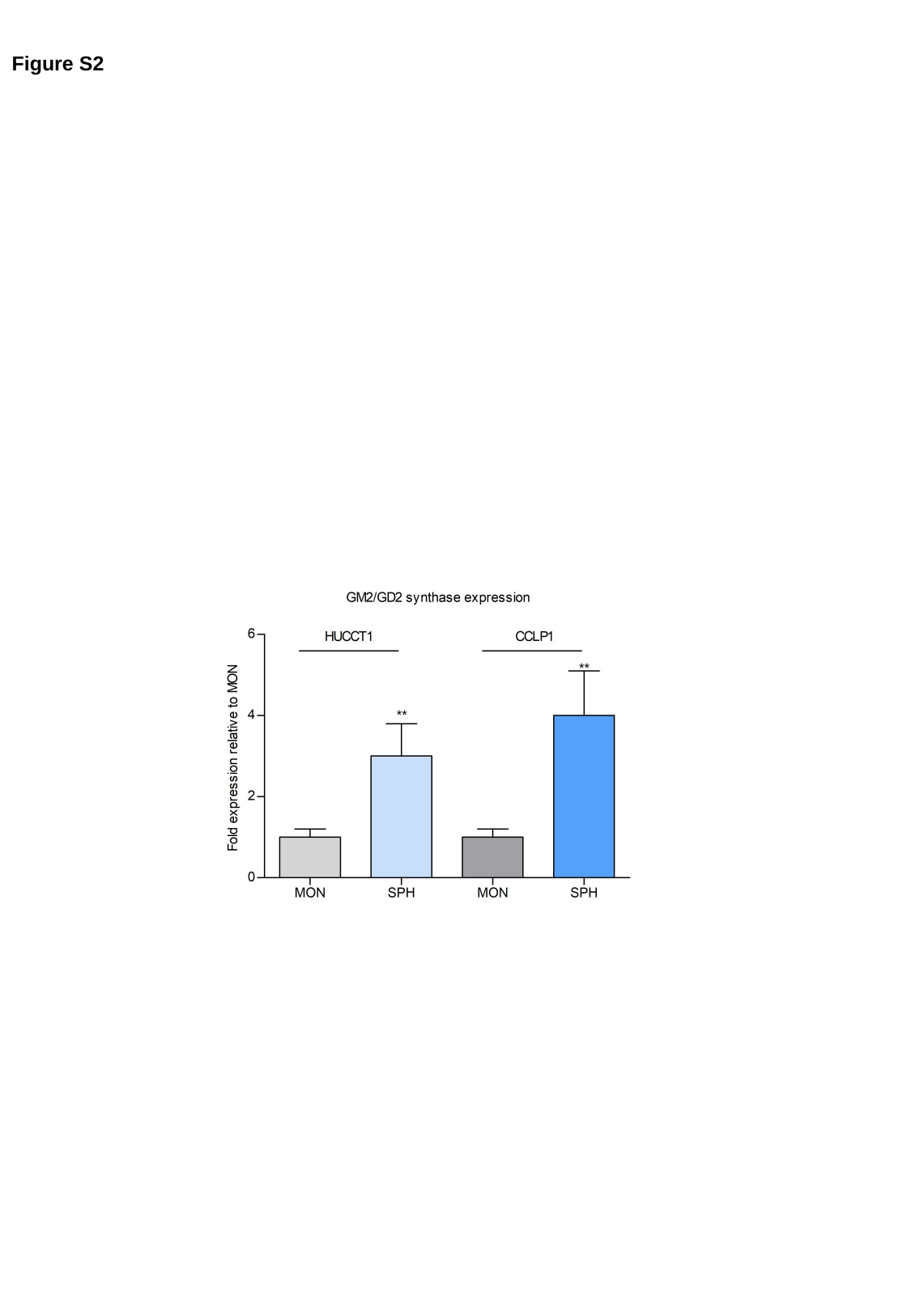

## Slide 3
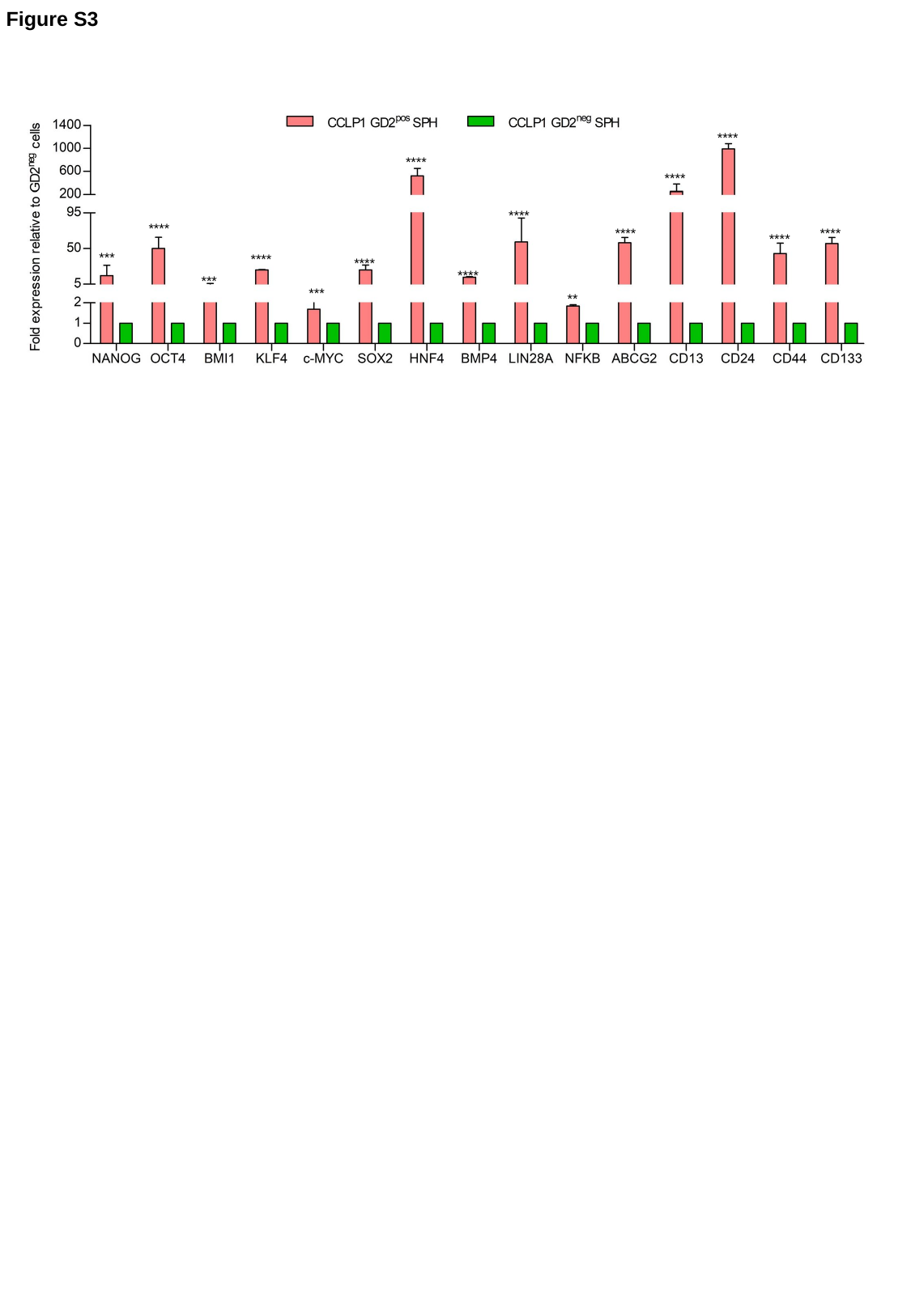

## Slide 4
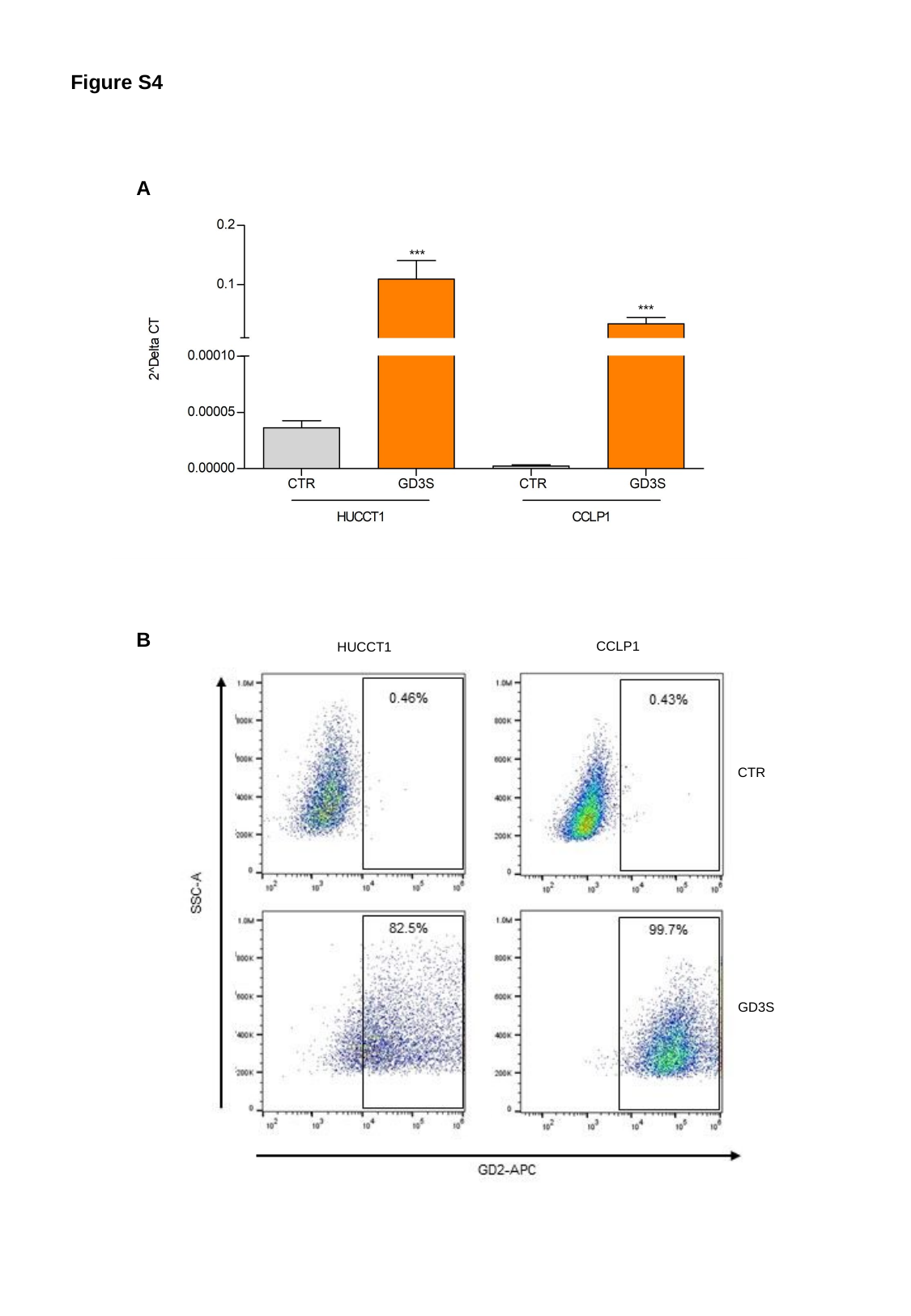

## Slide 5
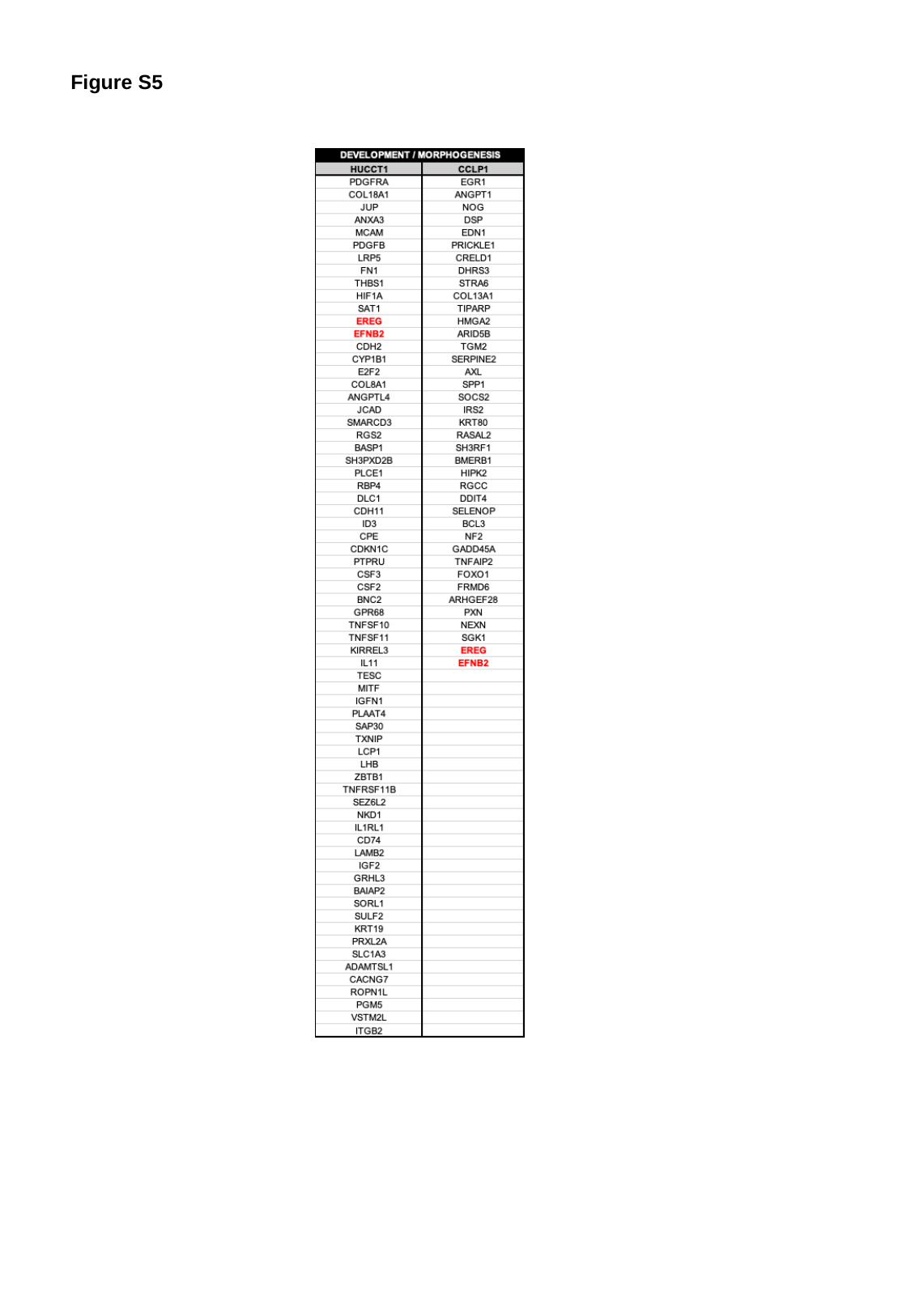

## Slide 6
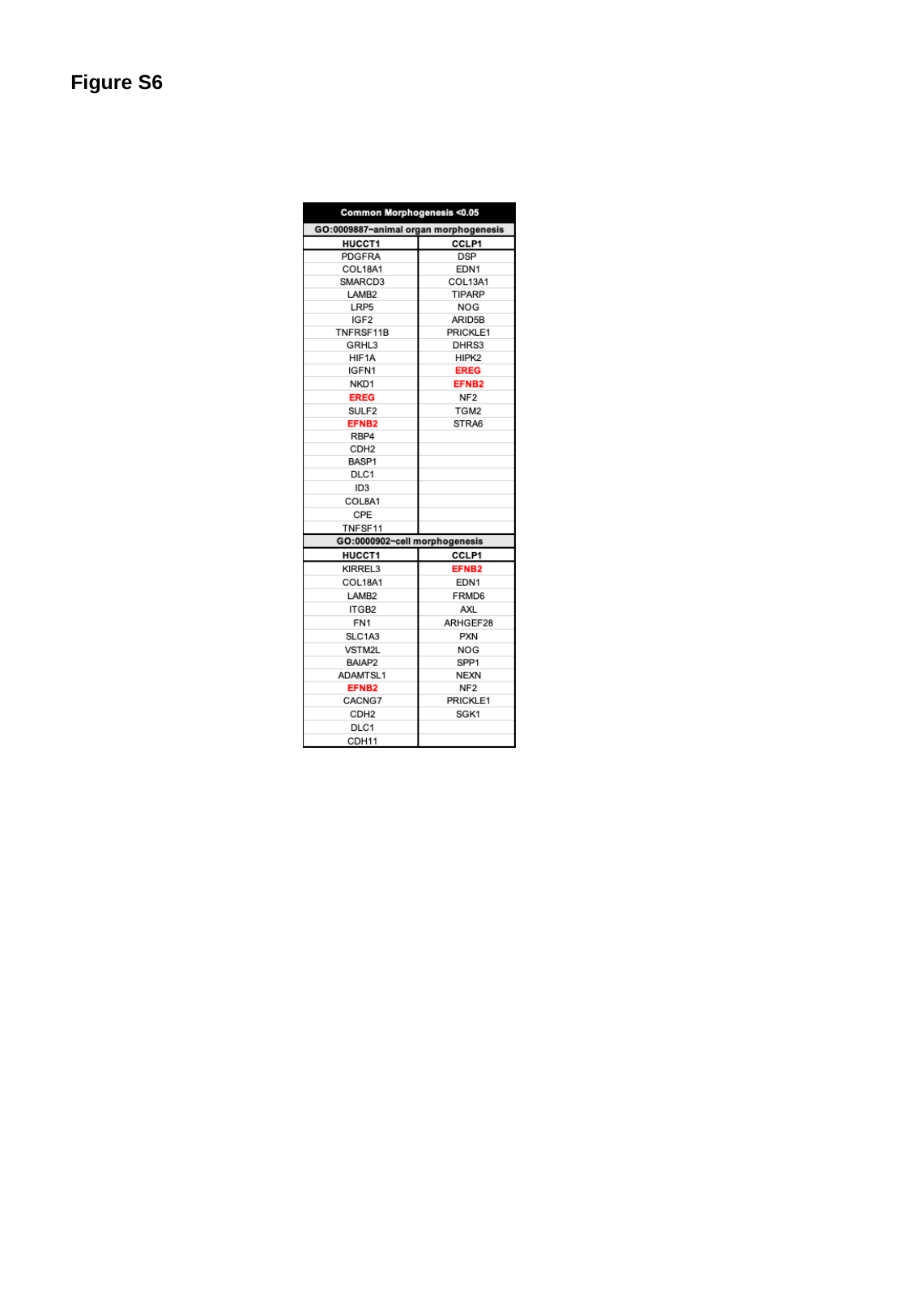

## Slide 7
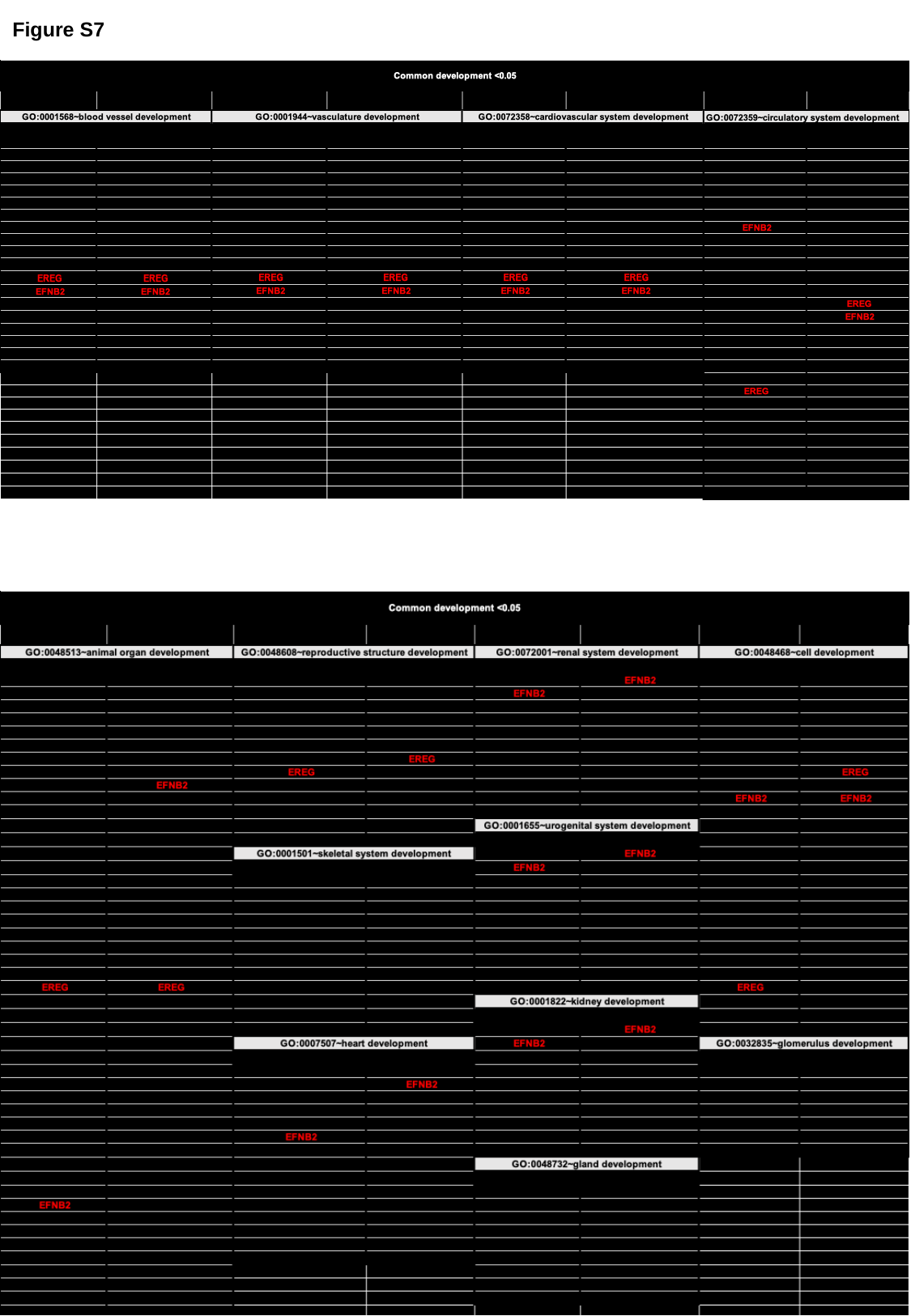

## Slide 8
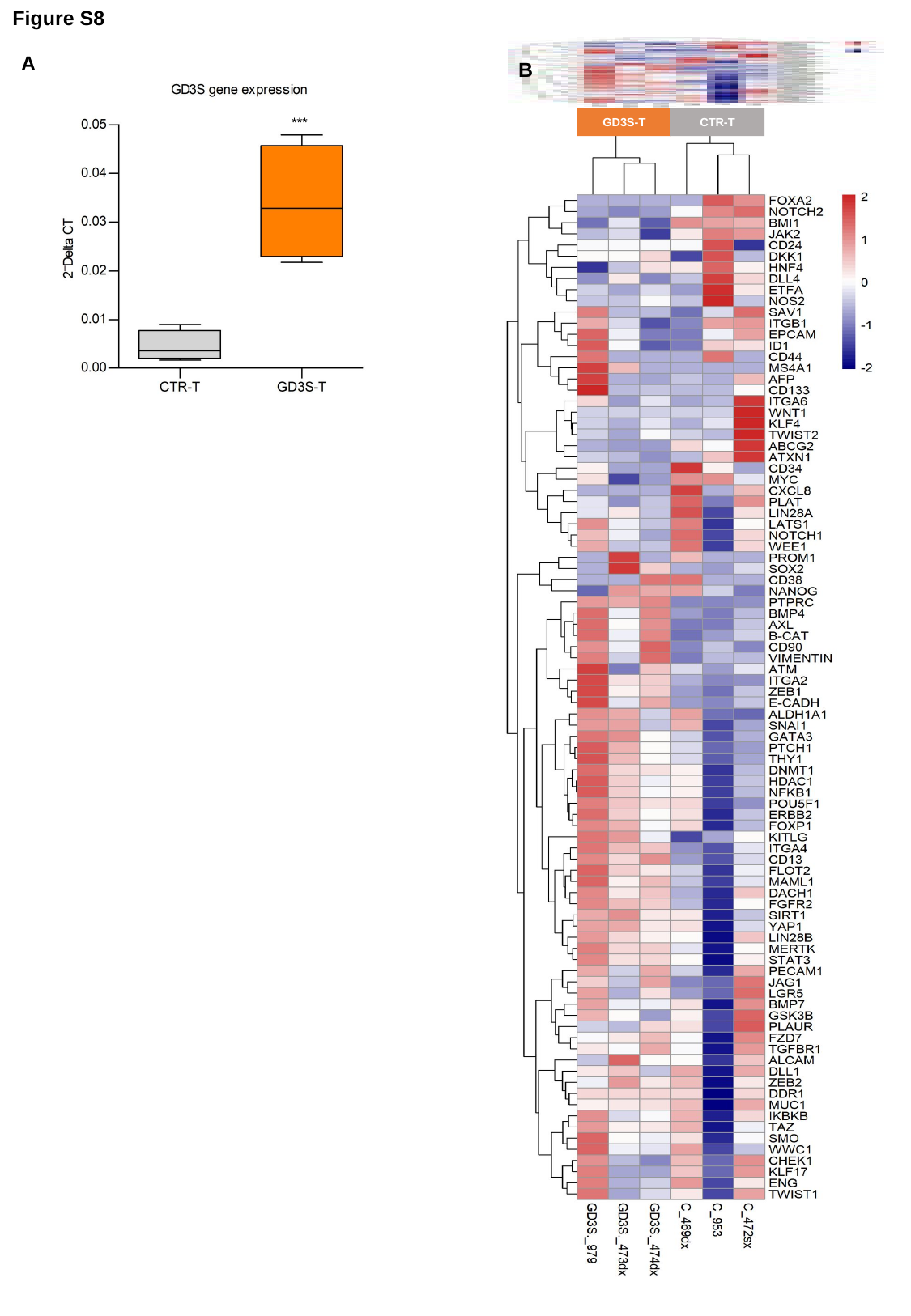

## Slide 9
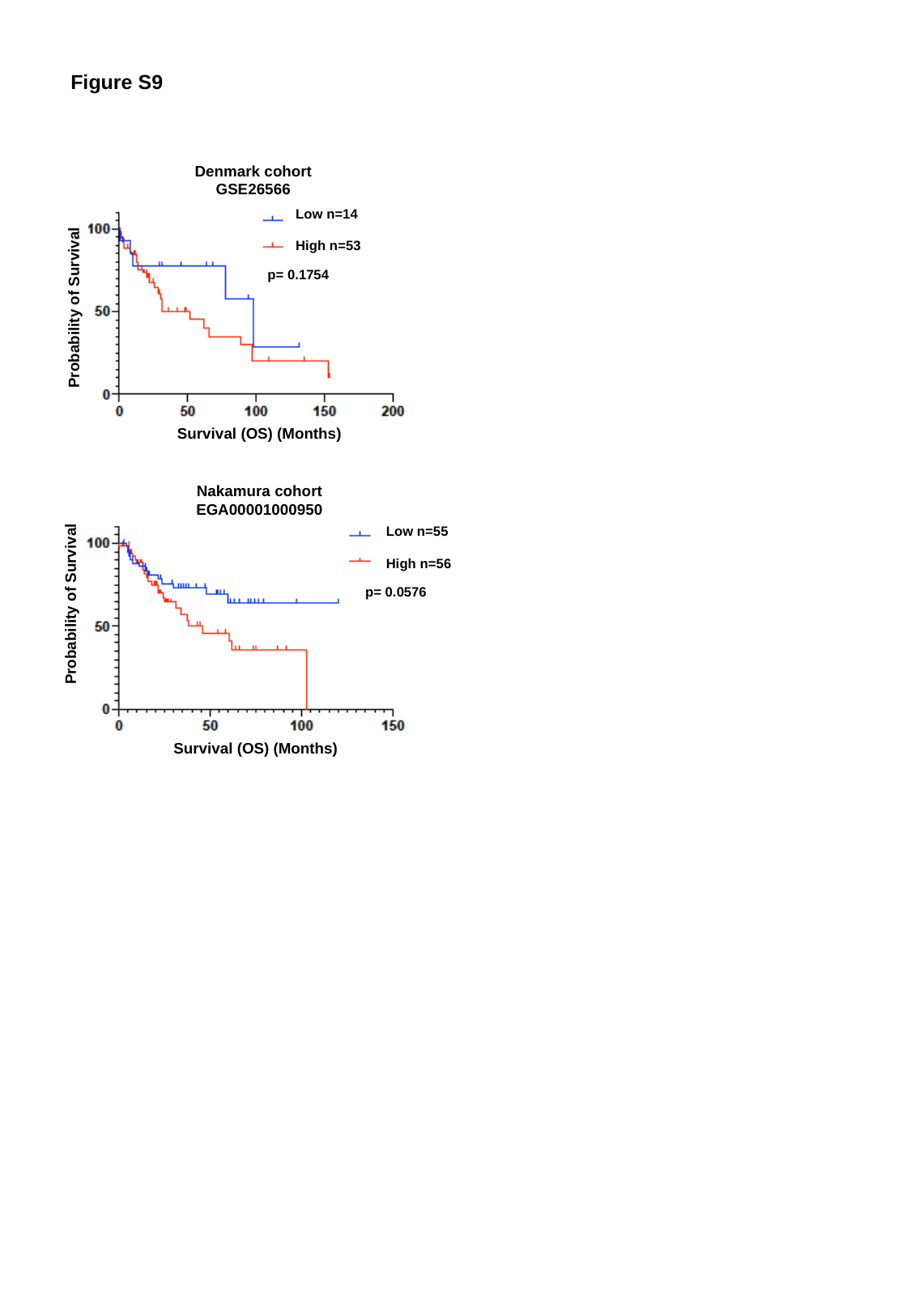

## Slide 10
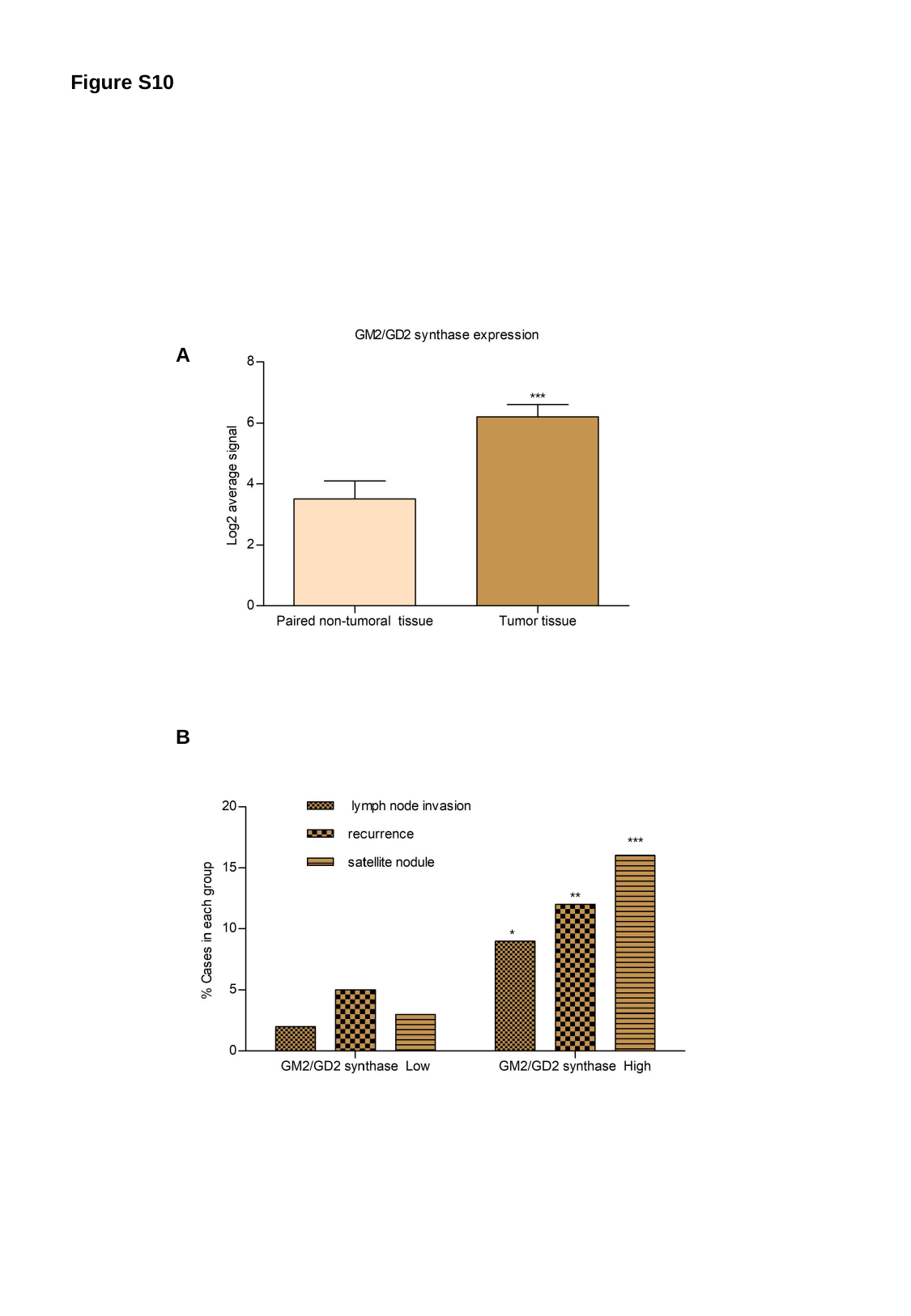

Supplement: Supplementary file 1 — Data S1. [file LIV-45-0-s001.zip › SupplementaryFigures-Final.pptx]
